# Functional differences of Munc18 isoforms reflect their affinities for the exocytosis site

**DOI:** 10.1007/s00018-026-06381-0

**Published:** 2026-08-08

**Authors:** Liangwen Liu, Santiago Echeverry, Muhmmad Omar-Hmeadi, Irina Česnokova, Per-Eric Lund, Jan Saras, Sebastian Barg

**Affiliations:** 1 Department of Medical Cell Biology, Uppsala University, Uppsala, Sweden; 2 Department of Animal Biosciences, Swedish University of Agricultural Sciences, Uppsala, 75007 Sweden

**Keywords:** Munc18, Syntaxin, Docking, Priming, Insulin granule, Exocytosis

## Abstract

The SM-protein Munc18 and its binding partner syntaxin are crucial for two distinct steps of regulated exocytosis, docking of secretory granules at the release site, and priming reactions that prepare docked granules for Ca^2+^ dependent fusion. Both proteins cluster simultaneously at the docking site seconds after a granule arrives at the plasma membrane. To study the mechanisms of these separate Munc18 functions, we generated a Munc18-1 knockout (1KO) and a double knockout of both Munc18-1 and Munc18-2 (DKO) in insulin-secreting cells, and quantified granule docking, exocytosis, and protein clustering at the docking site by TIRF imaging and capacitance measurements. In the 1KO, priming and exocytosis were lost, but granule docking remained intact. In the DKO, both priming and granule docking were impaired. Expression of either Munc18-1 or -2 rescued these defects, as did expression of mutants with weakened binding to syntaxin (EA and EK mutants). Munc18 clustered at granule docking sites, with apparent affinities decreasing in the order Munc18-1 > Munc18-1EA > Munc18-1EK > Munc18-2. Clustering of syntaxin-1 and − 3 at granules was impaired in both knockout lines. Finally, single molecule and imaging fluorescence recovery after photobleaching (FRAP) revealed that Syntaxin and Munc18 molecules were bound to each other, regardless of whether the proteins were clustered at a granule or not. We conclude that that Munc18-1 and − 2 molecules co-cluster with syntaxin at the release site and support docking and priming, and different affinities of the isoforms for the granule docking site contribute to the observed functional preferences.

**Supplementary Information:**

The online version contains supplementary material available at 10.1007/s00018-026-06381-0.

## Introduction

Regulated exocytosis of peptide hormones or neurotransmitters relies on the formation of a trimeric complex of SNARE proteins that bridges the vesicle and plasma membranes, and thereby facilitates fusion of the two membranes to allow release of the vesicle cargo. Assembly of this SNARE complex is facilitated by the S/M protein Munc18 that binds to the SNARE protein syntaxin with nanomolar affinity [1–4]. Interaction of Munc18 with syntaxin is required for exocytosis [5, 6], docking of secretory granules at the plasma membrane [7–9], and sorting of syntaxin to the plasma membrane [10, 11]. Both the granule docking step and subsequent priming reactions required for exocytosis are affected in Munc18-1 knockout models [5, 6, 10, 12] Defects in the function or expression of Munc18 isoforms are associated with type-2 diabetes [13–15], neurological disorders [16], and rare immunological diseases [17].

The Munc18-1 isoform expressed in neurons and pancreatic β-cells interacts with its binding partner syntaxin-1 via two distinct binding modes. In the first, Munc18 exerts a chaperone function by stabilizing the autoinhibitory “closed” conformation of syntaxin-1 [4, 18]. In this conformation, Munc18 arches around a four helix bundle formed by syntaxin’s SNARE domain and three additional helixes of its N-terminal Habc domain [4, 19]. This syntaxin-Munc18 complex precludes interaction with other SNAREs, but is likely the starting point for SNARE complex assembly [20]. The transition from closed syntaxin to the assembled SNARE complex is accommodated by conformational changes in Munc18 [21], and likely involves an intermediate complex in which Munc13 and Munc18 have a templating function for the participating SNARE proteins [2, 20, 22–24]. During this transition, a second binding mode is established, whereby the peripheral domain 1 of Munc18 binds to a short peptide in the extreme N-terminus of syntaxin (N-peptide) [2, 25–27]. Interfering with this transition prevents neurotransmission and reduces SNARE zippering [28], although the functional relevance of the N-peptide interaction is under debate [29–31]. Several mutations in Munc18-1 and syntaxin-1 selectively target either of the two binding interfaces, notably Munc18-1 K46E/E59K which affects binding to the closed conformation, and F115E/E132A that affects interaction with the N-peptide [11, 26, 32].

While Munc18 is in principle a soluble protein, binding to the transmembrane SNARE protein syntaxin results in most of Munc18 to be associated with the plasma membrane [33]. The two proteins accumulate together in ~ 0.1 μm wide clusters that contain 50–70 copies of syntaxin [34] and that are often situated beneath docked secretory granules [35–38]. Granule docking coincides with rapid and simultaneous clustering of Munc18 and syntaxin at the docking site, which in turn stabilizes docked granules at the plasma membrane [38]. Binding of Munc18 to syntaxin, likely to its auto-inhibited closed conformation, is required for both clustering of syntaxin and its function during granule docking [29, 37].

Mammalian cells express three Munc18 isoforms, Munc18-1, -2 and − 3, with Munc18-1 being involved in exocytosis of hormone-containing granules and synaptic vesicles [7, 10, 32, 39], while cytotoxic granules in mast cells and exocrine zymogen granules rely on Munc18-2 [40, 41]. Munc18-3 and its cognate SNARE syntaxin-4 function in constitutive exocytosis and trafficking of membrane proteins to the plasma membrane [42]. Munc18 isoforms tend to have a preference for specific syntaxin isoforms whereby Munc18-1 interacts with syntaxin-1 and − 2 [43], Munc18-2 interacts with syntaxin-3 and − 11 [44, 45], and Munc18-3 interacts with syntaxin-4 [2, 43]. However, there is considerable overlap in the expression patterns of Munc18 isoforms. For example, pancreatic β-cells express all three isoforms and there is evidence that all of these are involved in insulin granules exocytosis [46–49]. In contrast, neuropeptide secretion in chromaffin cells and neurons relies exclusively on Munc18-1 [50, 51].

In this work, we used a knockout and rescue approach in insulin secreting cells to analyze specific roles of Munc18-1 and − 2 isoforms for granule docking, priming, and protein clustering during insulin granule exocytosis. We report that both Munc18-1 isoforms can on their own rescue all Munc18 functions, although with varying efficiencies. Differences in the degree to which the two isoforms promote exocytosis reflect their relative affinities for the docking site, and lead to a preference for Munc18-1 during insulin granule priming.

## Results

### Knockout of Munc18-1 prevents insulin granule exocytosis

**Fig. 1:**
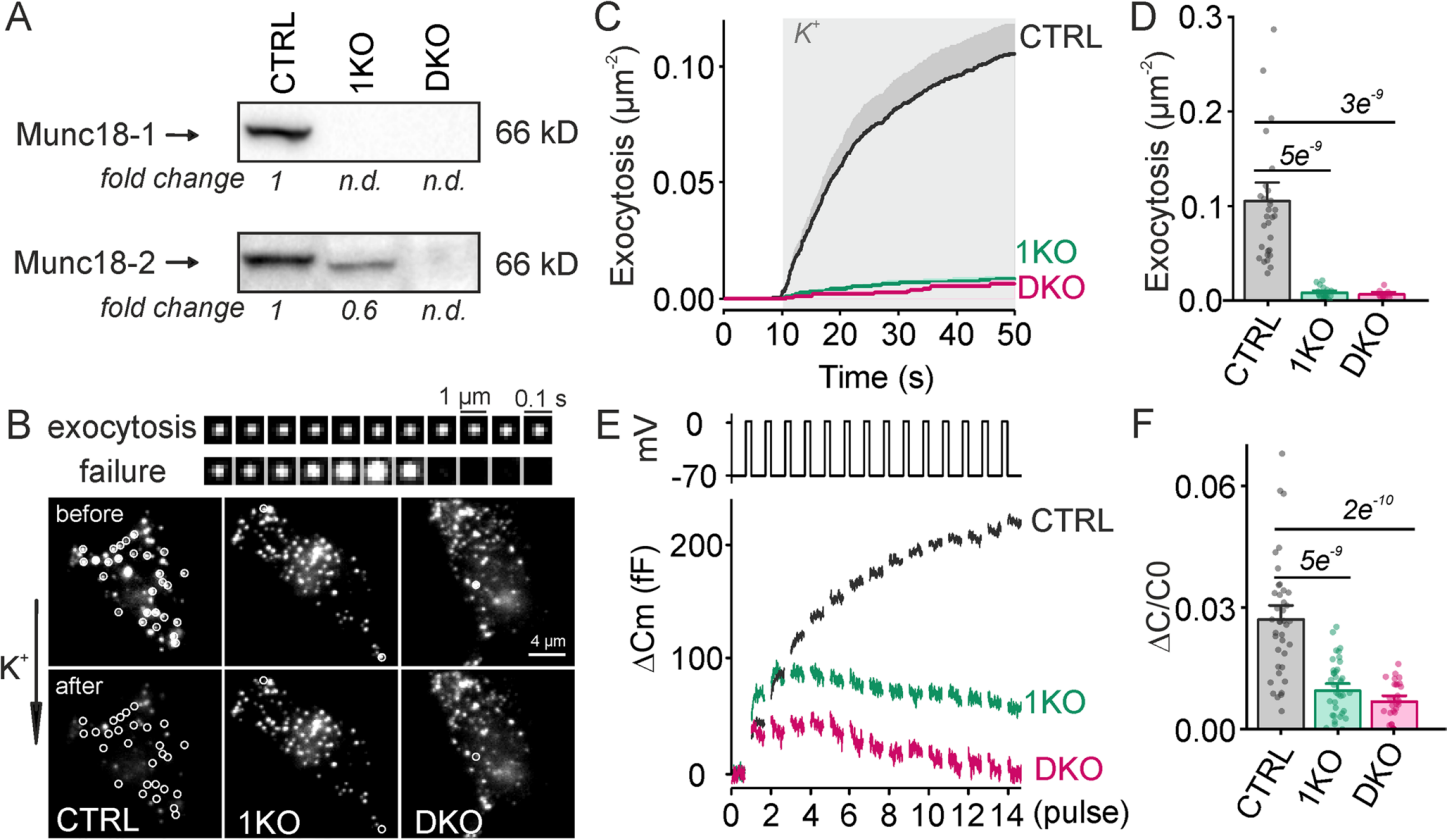
Knockout of Munc18-1 prevents exocytosis. **A **Immunoblotting analysis of Munc18-1 and Munc18-2 protein levels in control (CTRL), Munc18-1 single-knockout (1KO), and Munc18-1/2 double-knockout (DKO) INS1 cells. Cells were probed separately with anti-Munc18-1 (upper) and anti-Munc18-2 (lower) antibodies. Band intensities were normalized to total protein (stain-free) and expressed relative to CTRL (set to 1). Numbers below each lane (mean from N = 3 independent experiments) indicate fold change relative to CTRL; n.d., not detected. **B** TIRF micrographs of NPY-EGFP labeled insulin granules in CTRL, 1KO, and DKO INS1 cells. Cells were bathed in EC buffer containing 10 mM glucose, 2 µM forskolin, and 200 µM diazoxide, and imaged before and after stimulation with 75 mM K+ solution; exocytosed granules are marked with white circles. Scale bar: 4 μm. Time-lapse sequences illustrating individual granules undergoing exocytosis or remaining still without fusion (failure). Frame interval: 0.1 s. Scale bar: 1 μm. **C**-**D** Exocytosis of NPY-EGFP labeled insulin granules from CTRL (n = 30), 1KO (n = 19), and DKO (n = 11) INS1 cells. Elevated K+ was applied from 10–50 s. Cumulative time course plot in (**C**); shaded areas represent 1 SEM; total exocytosis and distributions in (**D**). **E**-**F** Representative whole-cell capacitance recordings (ΔCm) during 14 depolarizing pulses (-70 mV to 0 mV, 200 ms each) in (**E**). Quantification of capacitance changes from CTRL (n = 20), 1KO (n = 20), and DKO (n = 14) INS1 cells in (**E**). All data are presented as mean ± SEM. Statistical comparisons in (**D**) and (**F**) used the Kruskal–Wallis test with Dunn’s post hoc correction; p-values are indicated for the comparison against CTRL; p < 0.05 was considered statistically significant

To study the role of Munc18 during regulated exocytosis of dense core granules, we genome-edited insulin-secreting INS1 cells using CRISPR/Cas9 to generate knockouts of Munc18-1 (Munc18-1 KO), double knockout of both Munc18-1 and Munc18-2 isoforms (DKO); successful knockout in the clonal cell lines was confirmed by western blot (Fig. 1A). Granule docking and exocytosis were then studied in these cells by high-resolution TIRF microscopy with transiently expressed NPY-EGFP as granule marker. Exocytosis was stimulated by pressure ejecting an extracellular solution containing elevated K^+^ (75mM in presence of the K_ATP_-channel opener diazoxide) to cause cell depolarization and Ca^2+^ channel opening, and detected by the characteristic instantaneous loss of the fluorescence of individual granules (Fig. 1B). In control cells the depolarization resulted in exocytosis of 0.11 granules/µm^2^ during 40 s depolarization, while exocytosis in both Munc18-1 KO and DKO cells was reduced to about ~ 10% of that in control (Fig. 1C and D). This was confirmed by patch clamp recordings of membrane capacitance, in which exocytosis was triggered by trains of 14 depolarizations (200 ms, -70 mV to 0 mV, Fig. 1E and F). The combined capacitance increase was 212 fF in control INS1 cells. In contrast, Munc18-1 KO cells and DKO cells had robustly reduced exocytosis compared with control (Munc18-1 KO: -67%; DKO: -74%; Fig. 1E and F). Block of exocytosis was also observed by TIRF imaging when cells were instead stimulated with 10mM glucose (Fig S1). We conclude that insulin granule exocytosis requires expression of Munc18-1, while the additional effect of Munc18-2 knockout on exocytosis is relatively weak.

### Munc18 DKO leads to docking defect, with granule tethering intact

**Fig. 2:**
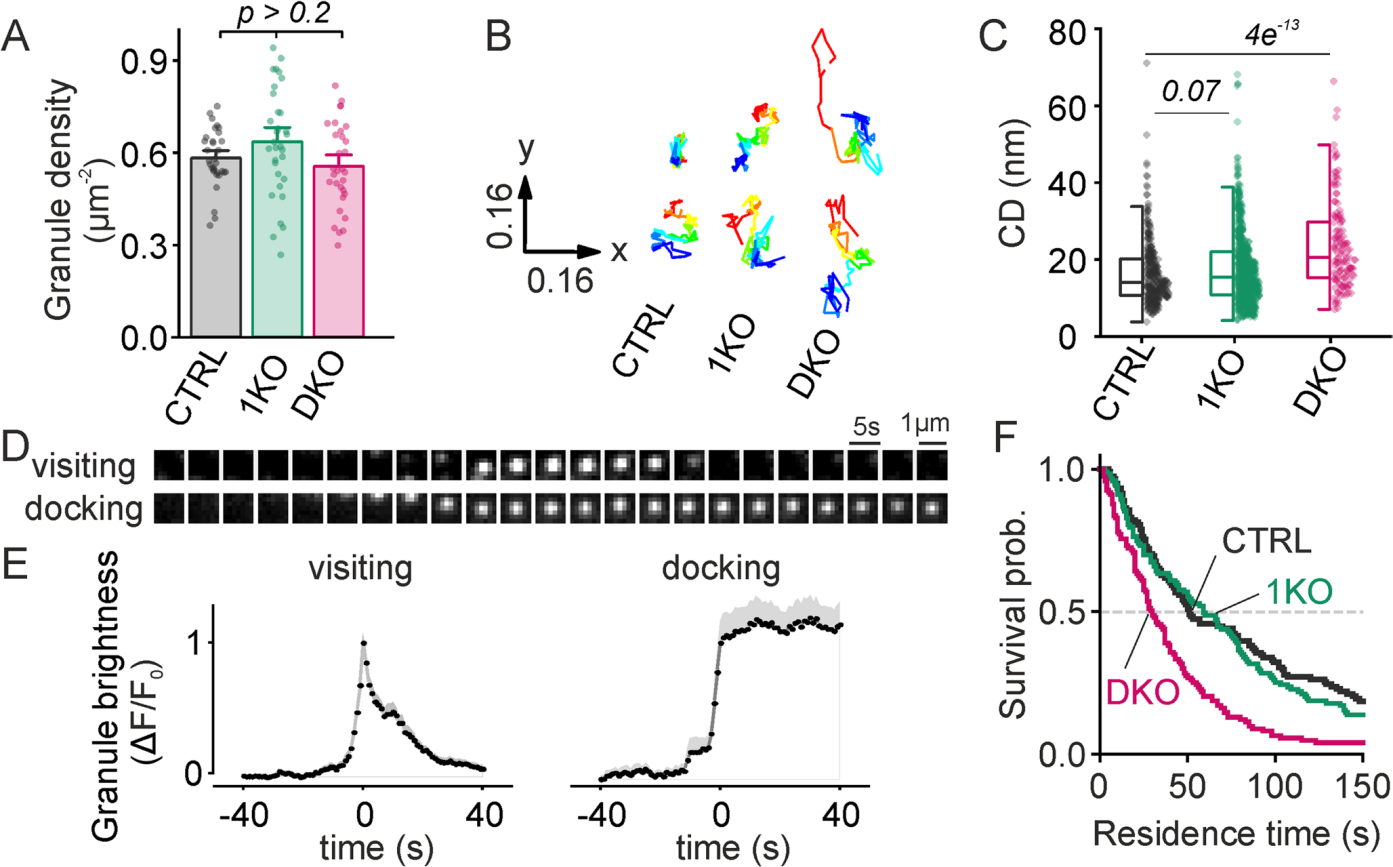
Double knockout of Munc18-1/-2 leads to mild docking defect. **A** Density of NPY-EGFP labeled granules visible under TIRF illumination in CTRL (n = 32), 1KO (n = 32), and DKO (n = 31) INS1 cells. Data are presented as mean ± SEM with individual cells shown as dots. **B** Examples of single granule trajectories for conditions as in A; two trajectories are shown for each cell type. No stimulation was applied. Scale: 0.16 μm, color indicates time. **C** Caging diameter analysis of granule trajectories from (**B**). Box plots display median with interquartile range (25th–75th percentiles); distribution of granules shown as dots. n = 317, 747, and 132 granules from CTRL (8 cells), 1KO (8 cells), and DKO (10 cells), respectively. **D** Examples of newly arrived granules demonstrating different behaviors: transient “visiting” (residence time ~ 30 s) versus stable “docking” (remaining throughout recording). Images acquired at 1 s intervals, displayed at 5 s intervals. No stimulation applied. Scale bar: 1 μm. **E** Mean fluorescence intensity traces (ΔF/ΔF0) for visiting events (residence time ≤ 30 s; 57 granules from 19 cells) and docking events (residence time > 60s; 21 granules from 9 cells). Traces are aligned to granule arrival at the plasma membrane (t = 0 s). Shaded areas represent 1 SEM. **F** Kaplan-Meier survival analysis of granule residence times following arrival at the plasma membrane. n = 118, 123, and 123 granules from CTRL (20 cells), 1KO (21 cells), and DKO (26 cells), respectively. The dashed horizontal line indicates the median survival probability (0.5). Statistical comparisons in (**A**) and (**C**) used the Kruskal–Wallis test with Dunn’s post hoc correction. Survival curves in (**F**) were compared using the log-rank test; p < 0.05 was considered statistically significant

To understand how granule docking depends on isoforms of Munc18, we quantified near-membrane granules by TIRF microscopy in control and knockout cell lines. The density of NPY-EGFP labeled granules was similar to control in all of the Munc18 knockout cell lines (Fig. 2A), suggesting that tethering of granules to the membrane still occurs in absence of both Munc18-1 and − 2, in contrast to previous observations in chromaffin cells. Although attached to the membrane, granules in the DKO appeared visually more mobile than granules in control cells. We therefore used particle-tracking analysis of near-membrane granules in movies (record 10 s at 10s^-1^/frame) to test how this lateral mobility depends on the presence of Munc18 (Fig. 2B). For most of the granules this mobility consisted of random excursions around a relatively constant mean location. We quantified this mobility as the granule caging diameter (CD, defined as a granule’s mean distance from its average position over 10 frames; see methods). Granules in control or Munc18-1 KO cells had on average smaller CD’s than those in DKO (CTRL vs. DKO: *p* = 4e^-13^; Kruskal-Wallis H test; Fig. 2C and representative tracks in Fig. 2B), with no significant difference between control and Munc18-1 KO cells (CTRL vs. Munc18-1 KO: *p* = 0.07). Since these differences suggest that lack of Munc18-2 leads to impaired granule docking, we also quantified granule residence times at the plasma membrane (5 min recordings at 1 frame*s^-1^; see examples in Fig. 2D and average granule brightness in Fig. 2E). Residence time distributions followed an exponential decay function with half times that were similar in control (median lifetime 53 s) and Munc18-1 KO (66 s, *p* = 0.4 vs. CTRL), but shorter in DKO cells (30 s, *p* = 3e^-7^ vs. CTRL, Fig. 2F). Similar results were obtained using the Zn²⁺-based granule marker ZIGIR, to exclude possible artifacts from using a genetically encoded marker (Fig. S2). The data suggest that granule attachment to the docking site is stabilized by Munc18-2, rather than by Munc18-1, and that weaker interactions remain in the absence of both proteins.

### Rescue of exocytosis and docking by Munc18 isoforms and mutants

**Fig. 3:**
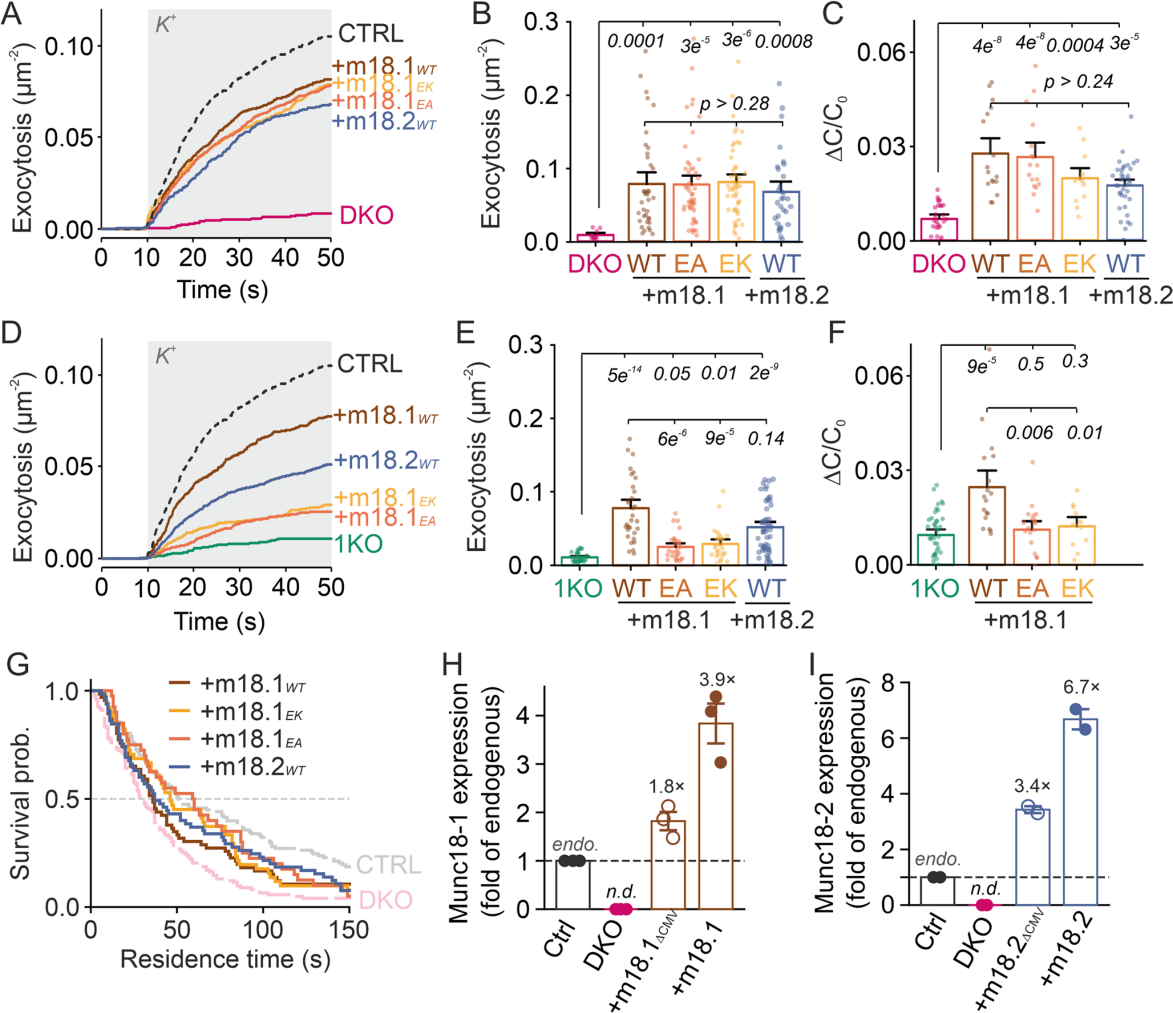
Rescue of exocytosis and docking defects in Munc18 knockout cells. **A**-**C **Functional rescue in DKO cells. (**A**) Cumulative exocytosis events during 40 s elevated K⁺ depolarization in DKO cells expressing NPY-EGFP alone or co-expressing mCherry-fused wild-type Munc18-1 (m18.1WT), Munc18-2 (m18.2WT), or mutants m18.1EA or m18.1EK. CTRL data (grey dotted line) reproduced from Fig. 1C for reference. (**B**) Total exocytosis events quantified from A. Data represent mean ± SEM with individual cells shown as dots. n = 10, 31, 38, 52, 53 cells from DKO, DKO+m18.1WT, DKO+m18.2WT, DKO+m18.1EA, and DKO+m18.1EK groups, respectively. (**C**) Membrane capacitance changes during a train of 14 voltage-clamp depolarizations. n = 14, 17, 38, 18, and 14 cells for the same conditions in (**A**). **D**-**F **Functional rescue in 1KO cells using the same experimental approaches as A-C. (**D**) Cumulative exocytosis. (**E**) Total exocytosis distribution. n = 30, 30, 52, 29, 28 cells from 1KO cells, rescue with Munc18-1, Munc18-2, Munc18-1EA, and Munc18-1EK. (**F**) Membrane capacitance changes. n = 20, 18, 20, 13 cells from corresponding conditions. **G **Kaplan-Meier survival analysis of granule residence times in DKO cells expressing indicated Munc18 constructs. n = 66, 65, 40, and 51 granules from DKO+m18.1WT (17 cells), DKO+ m18.2WT (14 cells), DKO+ m18.1EA (10 cells), and DKO+ m18.1EK (13 cells), respectively. CTRL (light grey dotted line) and DKO data (light pink dotted line) from Fig. 2F for reference. Rescue constructs DKO+m18.1WT, DKO+m18.1EK, DKO+m18.1EA, and DKO+m18.2WT compared to DKO (p = 0.03, 0.01, 0.003, and 0.02, respectively). Compared to CTRL (p = 0.03, 0.04, 0.1, and 0.02, respectively). **H**-**I **Quantification of Munc18-1 (H) and Munc18-2 (I) protein expression by immunoblotting in CTRL, DKO, and DKO cells transfected with the indicated constructs. Protein levels were normalized to total protein (stain-free) and corrected for transfection efficiency, then expressed relative to endogenous (endo.) levels in CTRL cells (dashed line, set to 1). Protein was not detected (n.d.) in DKO cells. The ΔCMV promoter drove near-endogenous expression (Munc18-1: 1.8×; Munc18-2: 3.4×), while the full CMV promoter produced supra-endogenous levels (Munc18-1: 3.9×; Munc18-2: 6.7×). Data are mean ± SEM; n = 3 and n = 2 independent experiments for Munc18-1 and Munc18-2 quantifications, respectively; individual values shown as dots. Statistical comparisons in (**B**) (**C**) (**E**) (**F**) used the Kruskal–Wallis test with Dunn’s post hoc correction. Survival curves in (**G**) were compared using the log-rank test; p-values are indicated for specific comparison; p < 0.05 was considered statistically significant

Since the DKO cells showed deficiencies in docking and exocytosis, they represent a useful model for studying Munc18 function using a rescue approach with mCherry-tagged Munc18 constructs. K^+^-stimulated exocytosis in DKO cells was almost completely rescued by expression of either Munc18-1 or Munc18-2, as assessed by TIRF microscopy (Fig. 3A-B) or capacitance measurements (Fig. 3C). Two well-studied point mutations with reduced binding affinity to syntaxin-1, K46E/E59K (Munc18-1_EK_) that prevent clasping around the 4-helix bundle of closed syntaxin, and F115E/E132A (Munc18-1_EA_) that prevent binding to syntaxin’s N-peptide [2, 11, 52–54] likewise rescued exocytosis in the DKO (Fig. 3A-C). In contrast, in Munc18-1 KO cells the degree of rescue of exocytosis depended on which Munc18 construct was expressed, in the order Munc18-1 > Munc18-2 > Munc18-1_EK_=Munc18-1_EA_ (Fig. 3D-F). Granule residence times, as a measure of stable docking, were partially rescued in the DKO by all four constructs, with little difference in efficiency (Fig. 3G). Protein quantification by Western blot indicated expression levels for the rescue constructs that were slightly above those of the endogenous proteins in wt cells, 1.8-fold for Munc18-1 and 3.4-fold for Munc18-2 after correction for transfection efficiency (Fig. 3H-I and S3). Since low expressing cells were selected during imaging (Fig S4A-B), actual levels were 2-3-fold lower and thus similar to endogenous expression levels. Use of a shorter ΔCMV promoter resulted in even lower expression levels that also rescued exocytosis (Fig S4C-D). Thus, endogenous levels of both Munc18 isoforms and the EA and EK mutants rescued docking and exocytosis in DKO cells. However, exocytosis in Munc18-1 KO cells was fully rescued only by Munc18-1.

### Clustering of Munc18 at the docking site

**Fig. 4:**
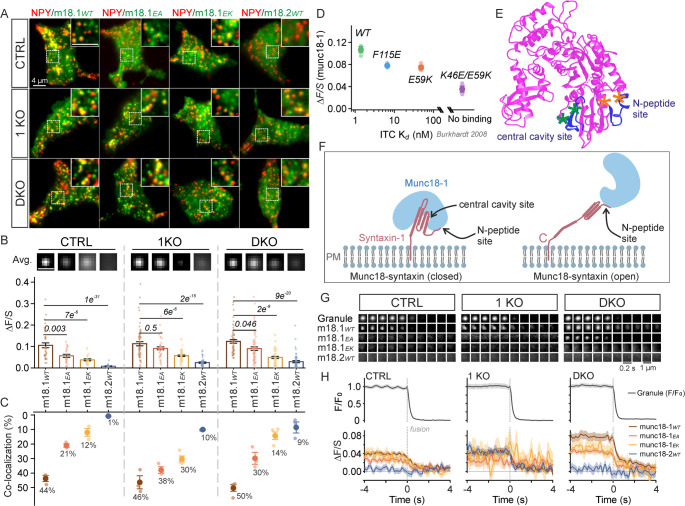
Clustering of Munc18 at the granule docking site. **A** Representative TIRF images of CTRL, Munc18-1 KO, and DKO INS1 cells co-expressing NPY-mCherry (granule marker, red puncta) with EGFP-tagged wild-type or mutant Munc18 constructs (green fluorescence), imaged in EC imaging buffer containing 10 mM glucose, 2 µM forskolin, and 200 µM diazoxide. Inserts show magnifications of the boxed areas. Scale bar: 4 μm. **B** Colocalization analysis of Munc18 constructs with NPY-positive granules. TIRF micrographs showing average Munc18 signal at NPY-positive puncta (upper, scale bar: 1 μm). Quantification of Munc18 fluorescence intensity at NPY granules (ΔF/S) shown below as mean ± SEM with individual cells as dots. For CTRL group: n = 897, 571, 518, and 845 granules from 55, 55, 48, and 81 cells expressing Munc18-1WT, Munc18-1EA, Munc18-1EK, and Munc18-2WT in CTRL cells, respectively. For Munc18-1 KO group: n = 950, 685, 961, and 533 granules from 60, 44, 59, and 39 cells, respectively. For DKO group: n = 801, 788, 656, and 671 granules from 61, 65, 58, and 60 cells, respectively. Statistical comparisons performed using Kruskal-Wallis one-way ANOVA followed by Dunn’s multiple comparisons test. p-values are indicated for the comparison against DKO/Munc18-1KO cells or with the expression of Munc18WT; p < 0.05 was considered statistically significant. **C** Frequency of Munc18 cluster colocalization with a granule (in %, mean ± SEM) from data in B. SEM is calculated from three independent experiments. **D** ΔF/S (Munc18-1 variants at granules) plotted against syntaxin-1/Munc18-1 variants binding affinity measured by Isothermal Titration Calorimetry (ITC; Kd values from Burkhardt, Pawel et al. 2008). **E** Structure of munc18-1 with highlighted binding areas for the SNARE bundle and the N-peptide of syntaxin-1 (Burkhardt, Pawel et al. 2008). Mutants used in this study are marked with asterisks. **F** Cartoon illustrating the two munc18-1 binding sites for syntaxin-1. **G** K⁺-stimulated exocytosis recorded in cells as in A. Time-lapse image sequences show averaged munc18 fluorescence spatio-temporally aligned to K⁺-stimulated exocytosis (t = 0 s). Frame interval: 0.2 s. Scale bar: 1 μm. H. Quantification of munc18-EGFP signal dynamics (ΔF/S ± SEM) in the same experiments as G, all data are spatio-temporally aligned to the moment of NPY-mCherry release shown in H (t = 0), Munc18 variants were separately paired with NPY-mCherry. Munc18-1WT-EGFP: n = 189, 178, 193 events from CTRL, Munc18-1 KO, and DKO cells; Munc18-1EA-EGFP: n = 72, 37, 171 events from CTRL, Munc18-1 KO, and DKO cells; Munc18-1EK-EGFP: n = 61, 48, 30 events from CTRL, Munc18-1 KO, and DKO cells; Munc18-2WT-EGFP: n = 54, 113, 66 events from CTRL, Munc18-1 KO, and DKO cells

Munc18 binding to the docking site was quantified in cells co-expressing a granule marker and EGFP-labeled Munc18 constructs (see examples in Fig. 4A), in two complimentary ways. First, we averaged small images of the Munc18 signal, each centered at the site of a docked granule (Fig. 4B, see methods). Averaging the Munc18-1 images for hundreds of granules revealed a bright spot at the center that indicated that this isoform was specifically recruited to granule docking sites (Fig. 4B: Avg. TIRF images). Dimmer spots were seen with the Munc18-1_EA_ and Munc18-1_EK_ mutants, and no spot was seen with Munc18-2 in control INS1 cells (Fig. 4B: Avg. TIRF images). Similar results were obtained with Munc18-1 KO cells and DKO cells (Fig. 4A and B). Accumulation of Munc18 at granules was also quantified by quantitative image analysis. We measured the Munc18-EGFP fluorescence specifically associated with the granule location (Δ*F*), and normalized this value by the fluorescence in a surrounding area that did not contain a granule (*S*; see methods and [55]. The resultant quotient Δ*F/S* can be interpreted as the apparent binding affinity of the tested protein to the docked granule location. Munc18-1 yielded Δ*F/S* values around 0.1, with no difference between control, Munc18-1 KO, or DKO cells (Fig. 4B: WT Δ*F/S* values for Munc18-2 were zero in control INS1 cells and around 0.03 in Munc18-1 KO and DKO cells (Fig. 4B), indicating relatively weak binding that increased in absence of Munc18-1. Δ*F/S* values for the Munc18-1_EK_ and Munc18-1_EA_ mutants were intermediate between Munc18-1 and − 2, and higher in both knockout cell lines compared with control INS1 cells (Fig. 4B: m18.1_EA_ and m18.1_EK_). Thus, association of the proteins with the granule docking site becomes progressively weaker in the order Munc18-1 > EA > EK> Munc18-2. Moreover, the values for Munc18-2 were higher in the Munc18-1 KO and DKO compared with control, suggesting that the presence of endogenous Munc18-1 interferes with binding of Munc18-2 to the docking site. The Δ*F/S* measurements were qualitatively similar to estimates by a (blinded) observer of the fraction of granules that co-localized with punctate Munc18 fluorescence (Fig. 4C). Moreover, Δ*F/S* measurements for Munc18-1 wt and mutants correlate with published protein binding constants obtained by ITC (Fig. 4D and [43]). When exocytosis was evoked by elevated K^+^, each of the four expressed proteins was rapidly lost from the release site during membrane fusion (Fig. 4G-H), similar to what is observed for syntaxin [38]. The data suggest that Munc18-1 competes with, and has a higher affinity for the granule-docking site than Munc18-2, and that both EA and EK mutants are able to accumulate at granules.

### Clustering of syntaxin in absence of Munc18

**Fig. 5:**
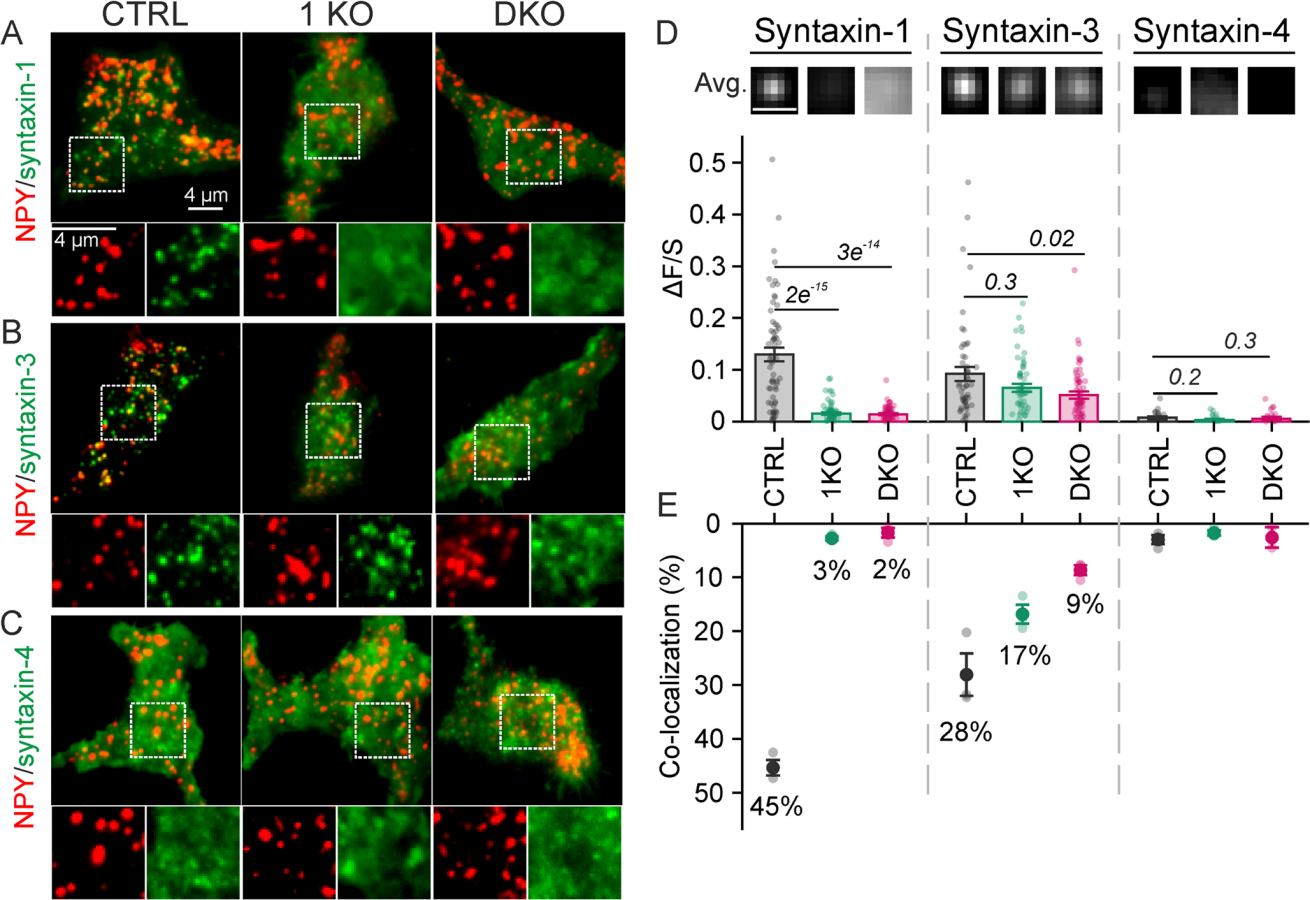
Clustering of syntaxin at granules. **A**-**C** Colocalization analysis of syntaxin-1, -3, and − 4 with insulin granules. (A-C) Representative TIRF microscopy images with magnified insets of CTRL, Munc18-1 KO, and DKO INS1 cells co-expressing NPY-mCherry (granule marker, red) and syntaxin-1-EGFP (**A**), syntaxin-3-EGFP (**B**), or syntaxin-4-EGFP (**C**). Scale bar: 4 μm. **D** Average syntaxin-1, -3, and − 4 signal labeled granules in images as in A-C (upper, scale bar: 1 μm). Lower panel shows quantification of syntaxin-1, -3, and − 4 fluorescence intensity at NPY granules (ΔF/S; mean ± SEM) with individual cell averages shown as dots. Syntaxin-1: n = 1248, 976, and 701 granules from CTRL (63 cells), Munc18-1 KO (68 cells), and DKO (69 cells). Syntaxin-3: n = 692, 544, and 758 granules from CTRL (52 cells), Munc18-1 KO (54 cells), and DKO (60 cells). Syntaxin-4: n = 1,098, 850, and 368 granules from CTRL (102 cells), Munc18-1 KO (95 cells), and DKO (40 cells). **E** Frequency of NPY-stained granules that colocalized with a syntaxin-1, -3, or -4cluster in images as in A-C (in %, mean ± SEM), SEM is calculated from three independent experiments. Kruskal-Wallis one-way ANOVA followed by Dunn’s post hoc test was used for (**D**). p-values are indicated for specific comparison; p < 0.05 was considered statistically significant

Munc18-1 co-clusters with syntaxin-1 at docked granules [29, 37, 38], which prompted us to quantify clustering of syntaxin isoforms 1, 3, and 4 at tethered/docked granules in the Munc18 knockout cell lines. Cells were co-transfected with C-terminally EGFP-labeled syntaxin isoforms together with the granule marker NPY-mCherry, and imaged by TIRF microscopy (Fig. 5). As reported previously, syntaxin-1 showed strong association with granules in control cells, seen as clusters that overlapped with granules and ΔF/S around 0.12, with 45% of positive co-clustered with insulin granules (Fig. 5A and D-E: CTRL). This association was almost completely lost in Munc18-1 KO and DKO cells (Fig. 5A and D-E). Association of syntaxin-3 with granules was overall weaker than that for syntaxin-1, but only moderately reduced in the DKO cell lines compared with control INS cells (Fig. 5B and D-E). Syntaxin-4 accumulation at granules was essentially zero in control and the knockout cell lines (Fig. 5C and D-E), suggesting that this isoform does not participate directly in insulin granule exocytosis. Traditional co-localization analysis by Pearson correlation confirmed that syntaxin-1 aggregates at granules, and that this was lost in both KO cell lines (Fig. S5). By the same method, colocalization of syntaxin-1 with Munc18-1 or Munc18-2 increased upon re-expression of the proteins in 1KO and DKO cells (Fig. S5). We conclude that Munc18-1 and − 2 promote syntaxin clustering at granule docking sites, and that loss of both isoforms disrupts the spatial organization of the exocytotic machinery.

### Munc18-1 and Munc18-2 bind to freely diffusing syntaxin-1 molecules

**Fig. 6:**
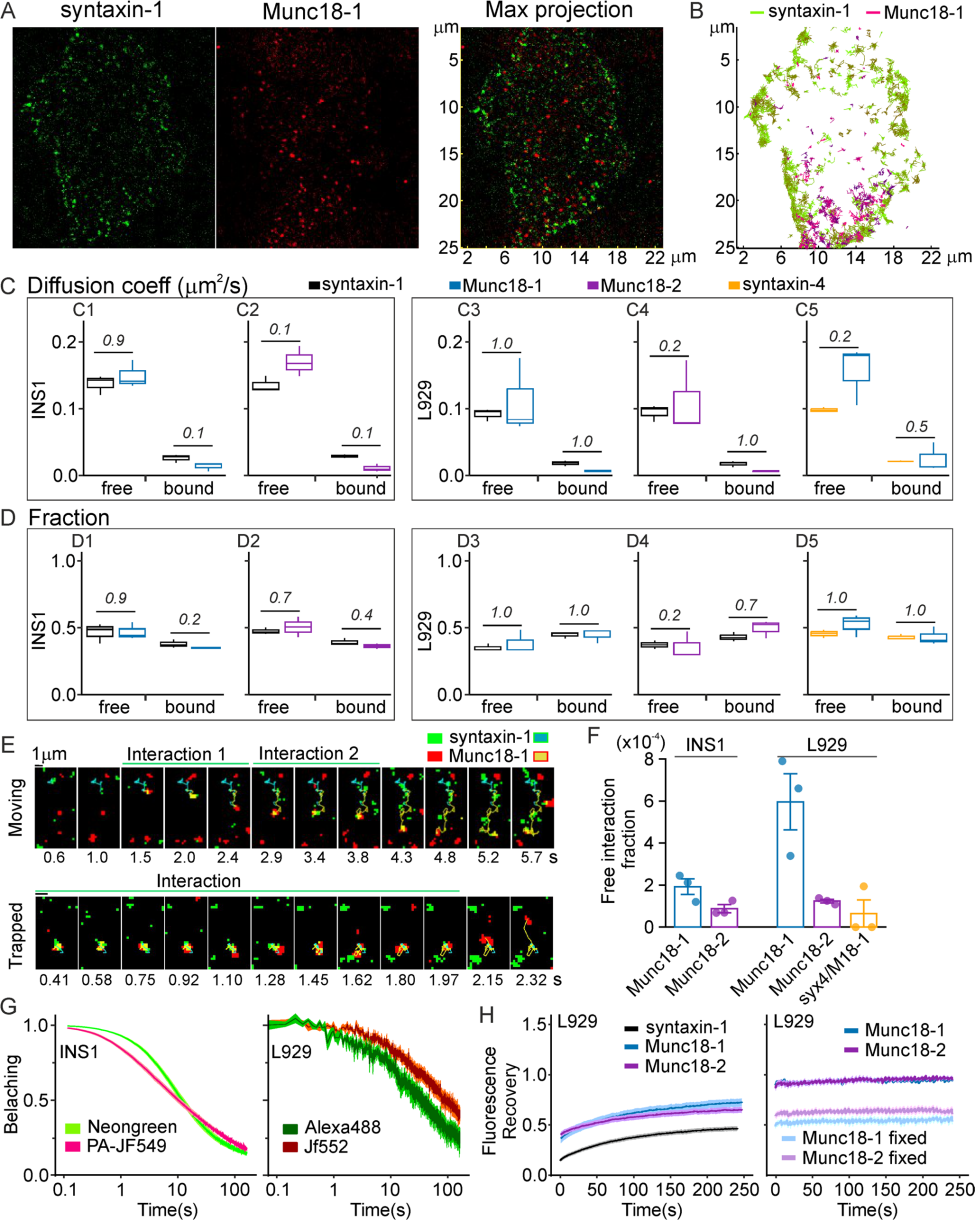
Single molecule imaging of syntaxin-1 and Munc18s. **A**-**B** Single molecule tracking setup and analysis. (**A**) Example image of a INS1 cell co-expressing JF-PA-549-labeled Munc18-1-HaloTag (red) and neonGreen labeled syntaxin-1 (green); Exposure time: 50 ms. (**B**) Example tracks obtained from 1000-frames recordings of the cell in A, showing syntaxin-1 (green) and Munc18-1 (red) trajectories. **C** Diffusion coefficients derived from single molecule tracks in free and bound state of Munc18-1 (n = 3 days, 153 videos, 93483 tracks in INS1 and n = 3 days, 128 videos, 60317 tracks L929 cells) co-expressed with syntaxin-1 (87077 tracks in INS1 and 51671 tracks in L929), Munc18-2 (n = 3 days, 149 videos, 23331 tracks in INS1 and n = 3 days, 118 videos, 45455 tracks in L929 cells), co-expressed with syntaxin-1 (89643 tracks in INS1 and 19502 tracks in L929 cells) and syntaxin-4 (n = 3 days, 112 videos, 15013 tracks in L929 cells) co-expressed with Munc18-1 (10635 tracks). Tracks from each day were fitted to a three-state kinetic model; each cell had 2–3 subsequent recordings. Mean values are shown as cube in boxes (Wilcoxon test). **D** Fraction of molecules in free and bound stated as in C (Wilcox test). **E** Examples of image sequences of syntaxin-1 (green molecule, cyan track) and Munc18-1 (red molecule, yellow track) tracks colocalized together. Upper example displays two molecules moving together and interacting two times. Lower example shows two molecules trapped together. **F** Relative frequency of observed uncaged protein interactions, the box indicated the median value. **G** Bleaching curve of neongreen and PA-JF-549 under the same recording conditions for INS1 cells (n = 17 cells), left panel. Right panel, bleaching curve of Alexa488 and JF-552 under the same recording conditions for L929 cells (n = 8 cells). **H** Mean fluorescence recovery ± SEM of L929 cells, munc18-1 (n = 17), munc18-2 (n = 18), and syntaxin-1, left panel. Right panel, mean fluorescence recovery ± SEM for L929 expressing Munc18-1 (n = 10) or -2-EGFP (n = 10) alive or formalin fixed (munc18-1 n = 6 and munc18-2 n = 7) L929 cells

We used single molecule imaging to understand the interaction between Munc18-1 and Munc18-2 with syntaxin-1 at the membrane. INS1 cells co-expressing Munc18-1-HaloTag or Munc18-2-HaloTag (labeled with PA-JF549) together with syntaxin-1-neongreen were imaged by TIRF microscopy (8ms/frame, Fig. 6A and B). After weak photo-activation, mobile diffraction-limited spots were observed in both color channels. These spots displayed typical quantal bleaching behavior under intense illumination (not shown), indicating that they represented single fluorescent proteins. Visually, single molecules of both Munc18-1/2 and syntaxin-1 diffused across the cell footprint, as expected for a protein associated with the plasma membrane (see example in Fig. 6A and E). Single molecules were tracked, and track length distributions were similar for the trans-membrane protein syntaxin-1 (τ_fast_ = 250 ± 1 ms; τ_slow_ = 632 ± 76 ms) as for the soluble Munc18-1 (τ_fast_ = 413 ± 24 ms; τ_slow_ = 1150 ± 105 ms) and Munc18-2 (τ_fast_ = 309 ± 11 ms; τ_slow_ = 723 ± 41 ms), suggesting that Munc18s remained membrane associated during the observation period. Due to bleaching (Fig. 6G) and blinking [56] of the fluorophores, these data likely underestimate the time a molecule is associated with the membrane. A three-state diffusion model (assuming free, granule-clustered, and immobile molecules) was fitted to tracks longer than 20 frames (1.16 s), and estimated diffusion constants that were nearly identical for labeled syntaxin-1 compared with co-expressed Munc18-1 or -2 molecules (Fig. 6C-D ①②, excluding rare immobile molecules < 2 nm^2^/s). Similar experiments were performed in L929 fibroblasts (that lack Ca^2+^-dependent exocytosis and endogenous syntaxin-1), by expressing Syntaxin1-SNAP/Alexa488 and Munc18-1/2-HALO-PA-JF-549/JF-552. Again, diffusion coefficients for syntaxin-1 and munc18-1 or -2 were similar (Fig. 6C-D ③④), but tended to be slightly smaller than those in INS1 cells. In contrast, the diffusion constant for free munc18-1 was twice that of syntaxin-4, when the two proteins were co-expressed (Fig. 6C-D ⑤), likely reflecting that the two proteins do not bind each other [43]. Despite the low labeling density in single molecule experiments, co-diffusion of Munc18s and syntaxin-1 could occasionally be observed directly in both cell lines (Fig. 6F). Expressed as fraction of all labeled molecules, munc18-1 was more often observed to co-diffused with syntaxin-1 than munc18-2, and co-diffusion of munc18-1 with syntaxin-4 was essentially absent (Fig. 6F). Double exponential functions fitted to the interaction time histograms revealed no differences between the proteins (τ_fast_ Munc18-1 0.29 ± 0.03s vs. Munc18-2 0.30 ± 0.03 s; τ_slow_ (Munc18-1 0.78 ± 0.09 s vs. Munc18-2 1.28 ± 0.23 s). Fluorescence recovery after photobleaching (FRAP) was used to complement the single molecule studies. When syntaxin-1 and munc18-1 or -2 were co-expressed in L929 fibroblasts, bleaching by intense TIRF illumination rapidly depleted the fluorescence of membrane associated proteins. The signal then slowly recovered (Fig. 6H left), which we interpret as slow, membrane bound diffusion of syntaxin-munc18 pairs from unbleached membrane above the bleached TIRF volume. Bleaching was not observed when Munc18-1 or -2 were expressed alone, presumably because bleached molecules were immediately replaced by unbleached molecules from the bulk cytosol. Indeed, bleaching (but not recovery) was observed when cells expressing munc18 alone were fixated (Fig. 6H right). Taken together, the data indicate that the majority of Munc18 molecules were bound to syntaxin-1, and that the two proteins either co-diffuse freely in the plasma membrane or are trapped in clusters.

## Discussion

We have revisited the role of the S/M protein Munc18 during regulated exocytosis, with a focus on its localization to granules and isoform-specific functions during the docking and priming of insulin granules. We report differences in the efficiency of Munc18-1 and − 2 isoforms for supporting exocytosis of insulin granules, where Munc18-1 is the preferred isoform for priming of insulin granules while both isoforms redundantly support granule docking. Although each isoform could rescue exocytosis in absence of the other isoform, the data suggest that the two isoforms preferentially serve separate functions during docking and priming. High-resolution imaging indicated that the apparent functional differences between the two isoforms are related to their relative affinity for the granule docking site, where they accumulate and promote the recruitment of syntaxin-1.

Insulin granule docking was essentially unaffected by single knockout of Munc18-1, indicating that either isoform can support docking at endogenous expression levels. In the double knockout, we found that the number of insulin granules at the plasma membrane was only mildly reduced, but the remaining granules showed decreased membrane interaction that manifested in both shorter residence times and increased motion at the membrane. The finding is consistent with relatively minor reduction of docked granules in Munc18-1 KO mouse β-cells [8, 37], but differs from mouse chromaffin cells where near-membrane granules were essentially absent in the Munc18-1 knockout. One reason for the discrepancy is that (still image) TIRF microscopy cannot distinguish between loosely tethered and docked granules, unlike electron microscopy. When we instead used particle tracking analysis of granules in live cells, tethered and docked granules were distinguishable. In addition, the presence of Munc18-2 in insulin secreting cells partially rescues docking in Munc18-1 KO cells [51], similar to what we report here for the DKO. The finding that the number of near membrane granules was unaffected in the DKO suggests that additional weaker tethering interactions exist between granule and plasma membrane that are independent of Munc18-1/2. Candidates for mediating Munc18 independent tethering include interactions between rab3/27 and its effectors RIM [57], rabphilin [58, 59], or granuphilin [60]. It is unlikely that Munc18c fulfils this function for insulin granules since it is not present at the docking site [29].

Knockout of Munc18-1 resulted in near complete block of insulin granule exocytosis, similar to what has been reported in PC12 and chromaffin cells [51, 61], where priming of granules is exclusively mediated by Munc18-1, while docking is supported by both Munc18-1 and − 2. However, the effect of Munc18-1 knockout on exocytosis could be partially rescued by over-expression of Munc18-2, suggesting that expression levels matter. The finding is in line with the relatively mild effects of Munc18-1 knockout on insulin secretion from mouse β-cells (that endogenously express Munc18-2) [8], and the reduction in “dead-end” docked granules after expression of Munc18-2 in chromaffin cells [62]. Differences between the Munc18 isoforms in supporting priming are therefore more likely due to differences in their expression levels and their affinities for the granule docking site, and may be exaggerated by the competition between Munc18-1 and Munc18-2 for binding to the release site ([51]; Fig. 4). Similarly, competition between Munc18 isoforms for syntaxin may underlie the finding that the weakly binding mutants (EA and EK) could rescue priming and exocytosis, but only in the absence of both Munc18-1 and Munc18-2. Clearly, both mutants retain the priming function of Munc18-1, albeit less efficiently. The functional differences between wildtype Munc18-1 and the mutants may therefore primarily reflect differences in their affinities of the Munc18-1 mutants for syntaxin-1 [53] and the docking site (Fig. 4 and ref [37]).

**Fig. 7:**
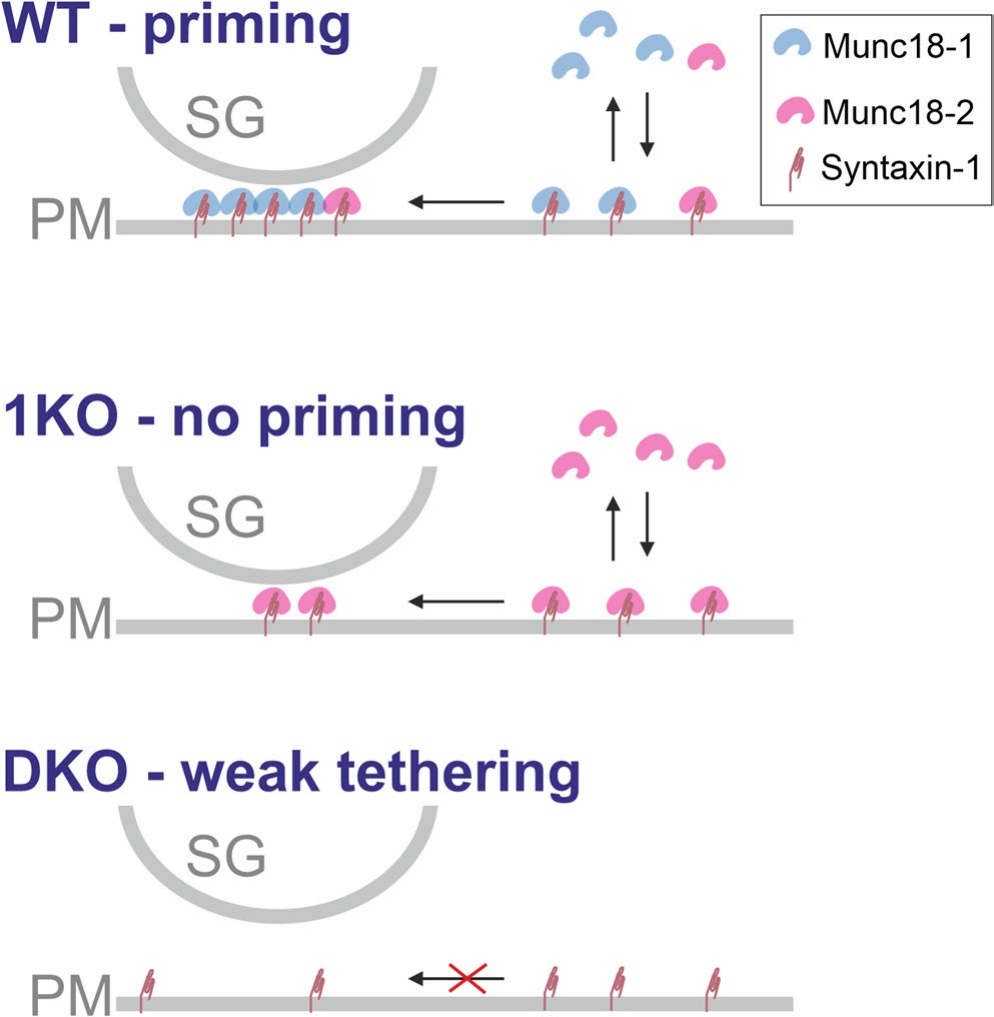
Munc18 affinity for the exocytosis site affects function. Cartoon summarizing the results. (**A**) In wt INS1 cells, both munc18 isoforms co-cluster with syntaxin-1 at the docked granule. The high concentration of syntaxin allows priming and exocytosis. Munc1. (**B**) In absence of Munc18-1 (1KO), fewer syntaxin-1 molecules are recruited to the release site, resulting in docked but mostly unprimed granules. (**C**) In absence of both Munc18 isoforms (DKO), docking is impaired due to the lack of munc18 and/or syntaxin at the release site.

We further show that both Munc18-1 and − 2 cluster at the site of docked granules, together with syntaxin-1 and − 3. This is consistent with earlier findings that syntaxin-1 and Munc18 are found at the docking site of insulin- and chromaffin cell granules [29, 37, 38]. Munc18-1 and syntaxin-1 arrive simultaneously at the docking site when a new granule docks, and clustering of the two proteins is critical for docking to succeed [38]. Knockout of Munc18-1 nearly abolished clustering of syntaxin-1 at docked granules, and reduced the accumulation of syntaxin-3, with little additional reduction in the double knockout (Fig. 5). This indicates that Munc18-1 is crucial for syntaxin-1 clustering at the granule, although both Munc18-1 and-2 isoforms are involved. In the absence of munc18-1, minimal amounts of syntaxin-1 at the release site may be sufficient to support docking, but not priming of the granule (Fig. 7). The mechanisms by which Munc18 helps to cluster syntaxin at the release site are not well understood, but involve binding of Munc18 to the closed form of syntaxin [29, 37], and an interaction between syntaxin/Munc18 and the rab effector proteins rabphilin or granuphilin on the granule membrane during docking [63–65]. Munc18-1 is also present on insulin granules [38], which may play a role during the induction of syntaxin/Munc18 cluster formation when a new granule arrives at the plasma membrane. Finally, our single molecule imaging data demonstrate that Munc18 is bound not only to clustered syntaxin beneath granules, but also bound to off-cluster syntaxin molecules that are diffusing individually in the plasma membrane (Fig. 6). This suggests that munc18 is already bound to the closed conformation of syntaxin when it arrives at the release site, rather than being recruited by binding to clustered syntaxin. Within granule-associated clusters, syntaxin may then serve two separate functions. First, to strengthen the physical interaction of the granule with the membrane, and second, to provide a reservoir and high local concentration of syntaxin molecules to facilitate SNARE complex formation (Fig. 7).

## Materials and methods

### Cell culture and transfection

Rat insulinoma INS1 cells (clone 832/13) [66] were cultured in RPMI 1640 medium (HyClone) containing 10 mM glucose, supplemented with 10% fetal bovine serum, streptomycin (100 µg/mL), penicillin (100 µg/mL), sodium pyruvate (1 mM), HEPES (10 mM), L-glutamine (2 mM) and β-mercaptoethanol (0.5 mM). Mouse fibroblast L929 cells were employed for single-molecule experiments and cultured in DMEM (Biowest) supplemented with 10% fetal bovine serum and penicillin (100 µg/mL). All cells were maintained at 37 °C in 5% CO₂. For imaging experiments, cells were plated onto poly-L-lysine-coated coverslips (22 mm diameter, Sigma-Aldrich), and transfected with DNA plasmids using Lipofectamine 2000 (Life Technologies). Imaging was performed 22–32 h post-transfection.

### Solutions

For imaging experiments, cells were bathed in extracellular solution (EC) containing (in mM): 138 NaCl, 5.6 KCl, 1.2 MgCl_2_, 2.6 CaCl_2_, 3 D-glucose, and 5 HEPES (adjusted to pH 7.4 with NaOH). In most experiments, the EC solution was supplemented with 10 mM glucose, 200 µM diazoxide (a K⁺-ATP-channel opener that prevents glucose-induced membrane depolarization), and 2 µM forskolin (Sigma) to increase exocytosis. Exocytosis was stimulated by depolarizing the cells with local application of a high K^+^ EC-solution (75 mM KCl equimolarly replacing NaCl), through computer-controlled pressure ejection from a glass capillary. Diazoxide and forskolin were not added in experiments that tested the granule residence time (Figs. 2D and F and 3G).

### Cloning

All expression plasmids were based on the pEGFP-N1 backbone (Clontech). NPY-EGFP and NPY-mCherry [55] were used as granule markers. Syntaxin-1-EGFP [55] was derived from rat syntaxin-1a (ORF of NM_053788) with linker sequence GVPRARDPPVAT-EGFP. Syntaxin-1-neongreen was constructed from rat syntaxin-1a with linker -VPRARDPPVAT-Neon (-Green). Munc18 constructs were derived from mouse Munc18-1 (ORF of NM_009295.2) [29]. Munc18-1-HaloTag consisted of Munc18-1 with linker RARDPPVAT fused to HaloTag. Munc18-2-HaloTag was constructed from human Munc18-2 (ORF of NM_006949.4) with linker TVPRARDPPVAT fused to HaloTag. Single point mutation (Munc18-1(E59K) and Munc18-1(F115E)), and double point mutations (Munc18-1_EK_ (K46E/E59K)-EGFP and Munc18-1_EA_ (F115E/E132A)-EGFP) were generated by site directed mutagenesis of Munc18-1-EGFP. For single molecule tracking, constructs for single molecules plasmid ΔCMV-Syntaxin-1-SNAPtag-P2A-Munc18-1-HaloTag was designed. This plasmid is driven by a minimal CMV promoter (nucleotides 94–487 of the CMV promoter/enhancer deleted. The expressed fusion protein is: Syntaxin-1-linker (GVPRARDPPVAT)-P2A-Munc18-1-linker (RARDPPVAT)-HaloTag). Plasmids ΔCMV-Syntaxin-4-SNAPtag-P2A-Munc18-1-HaloTag and ΔCMV-Syntaxin-1-SNAPtag-P2A-Munc18-2-HaloTag were constructed with a similar design (rat Syntaxin-4 sequence: ORF of NM_031125.2). All constructs were generated by PCR amplification of DNA fragments using Phusion or Q5 polymerases (New England Biolabs) followed by NEBuilder (New England Biolabs). Nucleotide sequence of constructs were verified by Sanger and/or Nanopore sequencing (Eurofins).

### CRISPR/Cas9 knockout cell

Chromosomal deletions in the Munc18-1 and − 2 genes were introduced into INS1 cells using CRISPR/Cas9 with the following target sequences: Munc18-1 (stxbp1): CCTGACTGAGGAGCAAGCATGGG and CTGCCAACTCGCTCCTGCCCTGG; Munc18-2 (stxbp2): ATGTCAGATATCTTGGCGGAGGG and CGGGTTGGAGTGAGGATGTGGG. These deletions removed most coding sequence for indicated Munc18. The CRISPR/Cas9 sequences were cloned into pSpCas9(BB)-2 A-Puro (PX459) [67] (RRID: Addgene_48139, a gift from Feng Zhang) and co-transfected with vectors using lipofectamine 3000 (ThermoFisher). Transfected cells were selected using 1 µg/ml puromycin (ThermoFisher) for 48 h. Single-cell clones were isolated and screened for Munc18-1, and Munc18-1/2 deletion using PCR performed on isolated genomic DNA. Clones identified as homozygote for deletions were chosen for experiments, except for the DKO where the selected clone has one Munc18-2 allele deleted and the other containing an insertion of one nucleotide at the 5` CRISPR target site. This insertion leads to a frameshift in the coding sequence at the amino terminus of M18-2.

### Immunoblotting

INS1 (control and knockouts) and plasmid transfected cells were lysed in RIPA buffer containing protease inhibitor (Cat. No. 11836153 001, Roche), centrifuged, combined with Laemmli sample buffer and denatured at 95 °C. Proteins were separated by SDS-PAGE (10% TGX™ Precast Protein Gel), transferred onto PVDF membranes, and immunoblotting with primary antibodies against Munc18-1 (rabbit anti-Munc18-1, Synaptic Systems #116002; 1:1000) or Munc18-2 (rabbit anti-Munc18-2, Synaptic Systems #116102; 1:2000), followed by HRP-conjugated goat anti-rabbit secondary antibody (Immun-Star GAR-HRP, Bio-Rad Laboratories #1705046; 1:20,000). Immunoreactive bands were detected using enhanced chemiluminescence substrate (Clarity Max Western ECL, Bio-Rad Laboratories), and quantified with a digital imaging system (Bio-Rad Laboratories). Band intensities were normalized for total protein of each sample using the stain-free technique.

### Whole-cell voltage-clamp recording

Patch-clamp recordings used an EPC-9 amplifier with PatchMaster v.2 × 90 software (HEKA Electronics). Borosilicate glass electrodes (2–4 MΩ resistance) were sylgard-coated and fire-polished. Cells were perfused at 0.4 ml/min with standard EC solution (composition as above) at 32 °C. Whole-cell voltage-clamp recordings used intracellular solution containing (in mM): 125 Cs-glutamate, 10 CsCl, 10 NaCl, 1 MgCl_2_, 0.05 EGTA, 3 Mg-ATP, 0.1 cAMP and 5 HEPES (adjust to pH 7.2 with CsOH). Exocytosis was evoked with 14 × 200 ms depolarizations from − 70 to 0 mV at 1.4 Hz. Membrane capacitance changes were quantified using the software lock-in module (30 mV peak-to-peak, 1 kHz).

### TIRF Microscopy

Microscopy was performed on a custom-built lens total internal reflection (TIRF) setup based on an AxioObserver Z1 inverted fluorescence microscope with a ×100/1.46 NA objective (Carl Zeiss). The excitation was from 3 DPSS lasers at 405, 491 and 561 nm (Cobolt) all passed through a cleanup quad-filter (zet405/488/561/640x, Chroma) and controlled with an acousto-optical tunable filter (AA Opto-Electronic). Excitation and emission light were separated using a beam splitter (ZT405/488/561/640rpc, Chroma). The emission light from two color channels was split and projected onto EMCCD camera (Roper QuantEM 512SC) using a dual-view beam splitter (Optical Insights) with a cutoff at 565 nm (565dcxr, Chroma) and emission filters (ET525/50m and 600/50m, Chroma). Scaling was 160 nm per pixel. For still images, the two-color channels were acquired sequentially, first with cells exposed to 491 nm (1mW) for 1 s (50 × 20ms average), immediately followed by 561 nm (0.2mW) for 100 ms. Movies were recorded with 100 ms exposure in stream mode (10 frames s^− 1^, K^+^-stimulated exocytosis) or 1 s exposure in stream mode (1 frame s^− 1^, docking experiments), and exciting simultaneously with 491 nm and 561 nm light. Alignment of the red and green channel was based on imaging 100 nm immobilized multifluorescent beads (Fluoresbite, Polysciences) at every recording session and implemented by a custom-written algorithm in Matlab (MathWorks, 2020) that translated, rotated, and resized the red image channel. Laser illumination intensity was routinely verified at the entrance into the microscope by a photodiode sensor (Thorlabs).

Single molecule, bleaching and FRAP experiments were carried in a similar TIRF microscope based on based on AxioObserver D1 microscope with an x100/1.46 objective (Carl Zeiss). Excitation was from a DPL at 561 nm and two MLD 473 nm and 405 nm lasers (Cobolt, Göteborg, Sweden), controlled by an acoustic-optical tunable filter (AOTF, AA-Optp, France) and using dichroic Di01-R488/561 (Semrock), the emission light was separated onto the two halves of a sCMOS camera (Prime 95B, Teledyne photometrics) using an image splitter (Dual view, Photometrics) with a cutoff at 565 nm (565dcxr, Chroma) and emission filters (FF01-523/610, Semrock; and ET525/50m and 600EFLP, both from Chroma). Scaling was110nm per pixel.

### Image quantification

Binding of fluorescently labeled proteins to the granule site was quantified as previously described [55]. Briefly, the goal was to separate granule-related fluorescence (on-granule, ‘bound’) from fluorescence not affected by the granule presence (off-granule, ‘free’). We quantified the average fluorescence in a circle (*c*) centered on the granule location and subtracted the average fluorescence in a surrounding annulus (*a*); the result Δ*F = c−a* represents fluorescence originating from granule-associated protein. Positive Δ*F* values indicate that the labeled test protein is concentrated at the granule, while negative values indicate exclusion. Off-granule fluorescence was obtained by subtracting out-of-cell-background (*bg*) from *a*; the result *S = a−bg* represents fluorescence originating from labeled protein that in unaffected by the granule. We then calculate *ΔF/S*, to adjust *ΔF* for differences in (local) expression level. Much like in binding assays of classical biochemistry, for many proteins the relationship of on- and off-granule fluorescence follows a simple one-site binding model in the form ΔF=(B_max_*S)/(Kd + S). The initial slope (Δ*F/S* for *S*<<*K*_*d*_) can be interpreted as the apparent binding affinity (*B*_max_*/K*_*d*_) of the tested protein to the granule site. In addition, the fraction of vesicles that were colocalized with a protein of interest was estimated visually. For every vesicle identified in the ΔF/S analysis, a script written in MetaMorph presented an investigator with 21 × 21 pixel area of the protein of interest that was centered on the vesicle location and autoscaled. The investigator then made a yes/no decision whether punctate fluorescence was present in the center of the presented area. Granule density was determined using ImageJ. Exocytosis events were found manually as granule events with an obvious change in the fluorescence from the pre-exocytosis baseline followed by sudden loss of the signal. Local Pearson correlation coefficients in Fig. 4C were calculated using a custom-written ImageJ macro. Individual syntaxin clusters were identified and required to be separated by at least 3 pixels (480 nm). For each cluster, the Pearson correlation coefficient between the two channels was measured within a 5 × 5-pixel square region (800 nm × 800 nm) centered on the syntaxin cluster position.

### Tracking and trajectory analysis for granules and single molecules

Insulin granule movement was tracked using TrackNTrace [68]. Spots representing granules were identified in each frame using the “Wavelet filter” plugin (scaling factor = 2, spline order = 3, detection radius = 2, detection threshold = 0.80), and refined using the “TNT filter” plugin (PSF = 1.3 pixels). Spot locations were linked into tracks using “u-track” (minimum track length of 10 frames, granules and single molecules respectively, no gaps, search radius 6 pixels). Granule caging diameters were calculated from 10 s long tracks, as the mean displacement of the granule position between consecutive 1s long segments. For survival curve analysis of insulin granule marked with ZIGIR (Fig. S2), a convolutional neuronal network (CNN), in MATLAB, was trained to identify the tracks belonging to single granules well dispersed from its neighbors (~ 92% accuracy). Single granule tracks lengths were fit to a proportional hazard’s regression model in R (library “survival”, coxph) to check for significative differences.

Single molecules were recorded in INS1 or L929 cells expressing low density syntaxin1-neongreen/SNAP-Alexa488 and low labelled Munc18-1 or -2- HALO-PA-JF-549/JF-552, during 1000 frames (58 ms/frame). When PA-JF-549 was used, photoactivation was adjusted to optimize fluorophore density in the cell footprint. Single molecules were tracked using the TrackNTrace framework; particle locations were linked into trajectories using the u-Track algorithm [68]. Diffusion coefficients were calculated for all tracks longer than 20 frames with Trackart [69]. Tracks were divided in three population (free, bound, immobile) by their cumulative probability distribution square displacement and the immobile fraction was excluded (< 20%, diffusion coefficient of <2nm^2^/s). A homemade MATLAB script was used to identify pairs of molecules colocalized in time and space. Interaction times of single munc18 and syntaxin molecules were identified as track segments during which the discrete Fréchet distances was less than 110 nm + localization error. Spurious interaction was estimated by randomized molecule locations of one of the channels within the cell area. Interaction tracks were discarded when their track length was equal or shorter than the longest interaction track in the randomized control (0.55s). Particles were classified as trapped or free moving, using the method in Lanoiselée et al. [70]. Single molecules localization error was estimated by a linear square method [71] and the average of the median localization error for the molecules was: Munc18-1-PA-JF549 38.97 ± 8.1 nm, Munc18-1-JF552 24.7 ± 5.6 nm, Munc18-2-PA-JF549 28.9 ± 8.6 nm, Munc18-2-JF552 30.5 ± 6.4 nm, syntaxin-1-neon-green 59.1 ± 2.0 nm, syntaxin-1-Alexa488 60.3 ± 2.7 nm, and syntaxin-4-Alexa488 61.3 ± 0.6 nm.

### Bleaching measurements

To measure the bleaching timecourse of fluorophores in single molecule experiments, INS-1 cells expressing Syntaxin1-neongreen and life-actin-Halo labelled with PA-JF-549 (*n* = 17 cells) or Syntaxin-4-Alexa488 and Munc18-1-JF552 (*n* = 8 cells) were fixed with 4% PFA and then imaged with settings as for single molecule recordings. After correction for background (excitation off), double-exponential decay function were fitted to the data to obtain fluorophore life times: Neongreen (τfast = 12 s ± 0.7 SEM, a fraction 71% ±1.8 SEM, τslow = 248 s ± 42 SEM), PA-JF549 (τ_fast_ = 10.5 s ± 1.2 SEM, a fraction 57% ± 1.9 SEM, and a τ_slow_ = 163 s ± 13 SEM), JF-552 (τfast = 18 s ± 2.2 SEM, a fraction 33% ±1.9 SEM, τslow = 265 s ± 35 SEM), and Alexa488 (τfast = 21 s ± 7.4 SEM, a fraction 57.2% ±5.9 SEM, τslow = 155 s ± 39 SEM).

### Fluorescence recovery after photobleaching

INS1 or L929 cells expression syntaxin1-neongreen and munc18-1 or -2-HALO-JF552 were used for the FRAP experiments. Initial cell fluorescence was measured with 0.5mW laser power (473 nm and 561 nm) at 100ms exposure time. Photobleaching was induced by 10mW laser power during 60s. Fluorescence recovery was measure with 0.5mW laser power at 100ms exposure time, initially by intervals of 0.5–2 s until 8s, and then by intervals of 5 s until 250s. Laser activation was intercalated throughout the entire protocol to avoid bleed through from the green to the red channel. The fluorescent recovey curve was then fitted with the formula Intensity_0_+amplitude_Recovery*(1-exp(-seconds/ τ)) and τ were used for statistical.

### Statistics

Experiments were conducted with at least 3 independent replicates. Data are presented as mean ± SEM unless otherwise stated. Data normality was assessed using the Shapiro-Wilk test. Multiple group comparisons were performed using Kruskal-Wallis H test followed by Dunn’s post-hoc test with Bonferroni correction. For two-group comparisons, paired Student’s t-test or Wilcoxon signed-rank test was used (as appropriate). A p-value < 0.05 was considered statistically significant.

## Supplementary Information

Supplementary Material 1.

Supplementary Material 2.

Supplementary Material 3.

Supplementary Material 4.

Supplementary Material 5.

Supplementary Material 6.

Supplementary Material 7.

## Data Availability

Raw data are available on request from the lead author.
