# Supplementary material for: Functional differences of Munc18 isoforms reflect their affinities for the exocytosis site
Source: Cell Mol Life Sci. 2026 Aug 8;83(1):298. doi: 10.1007/s00018-026-06381-0 (PMC13452794; doi:10.1007/s00018-026-06381-0)

Fig 1A_western blot

Stain free gel (10%)

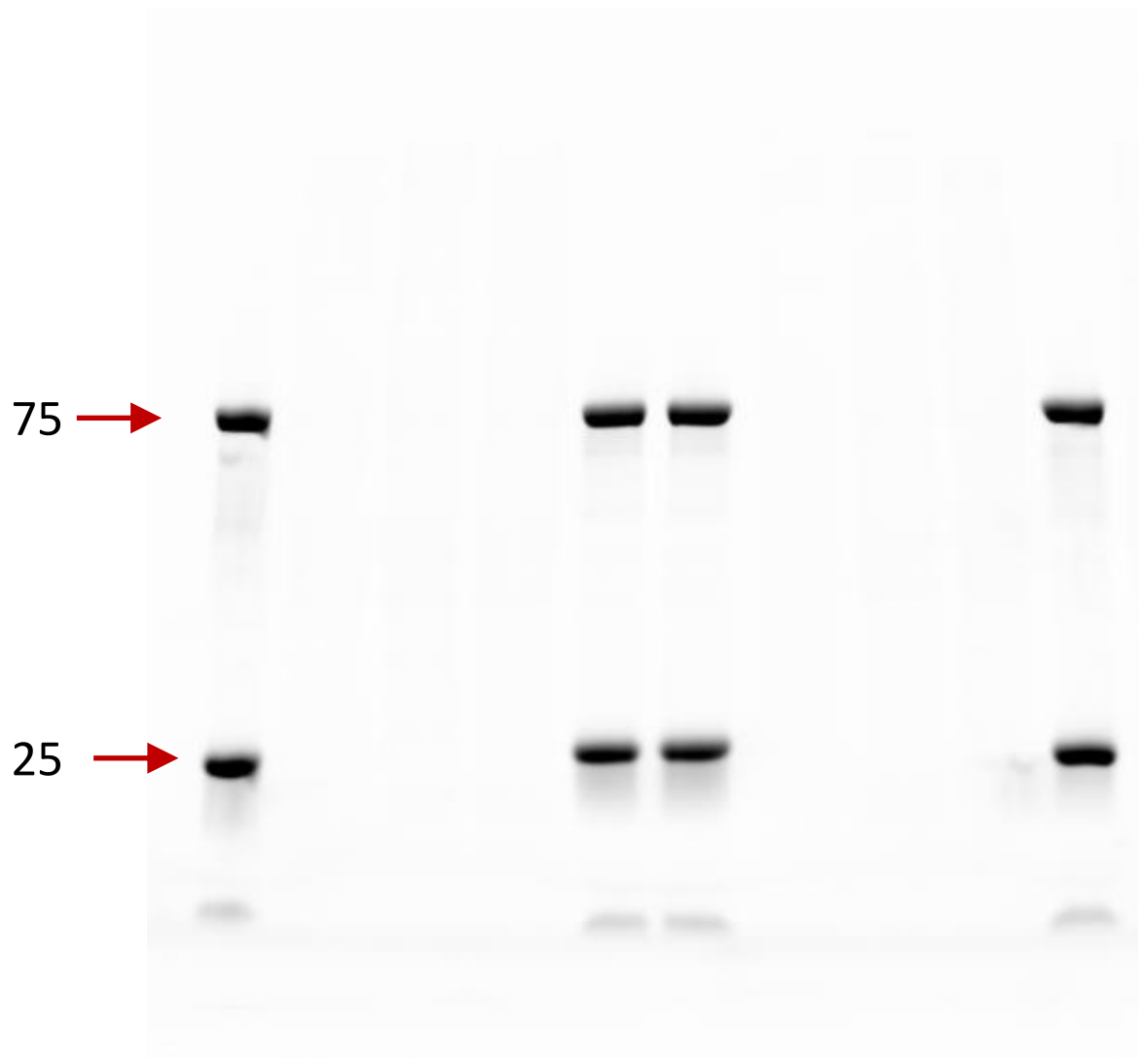

Chemiluminescence

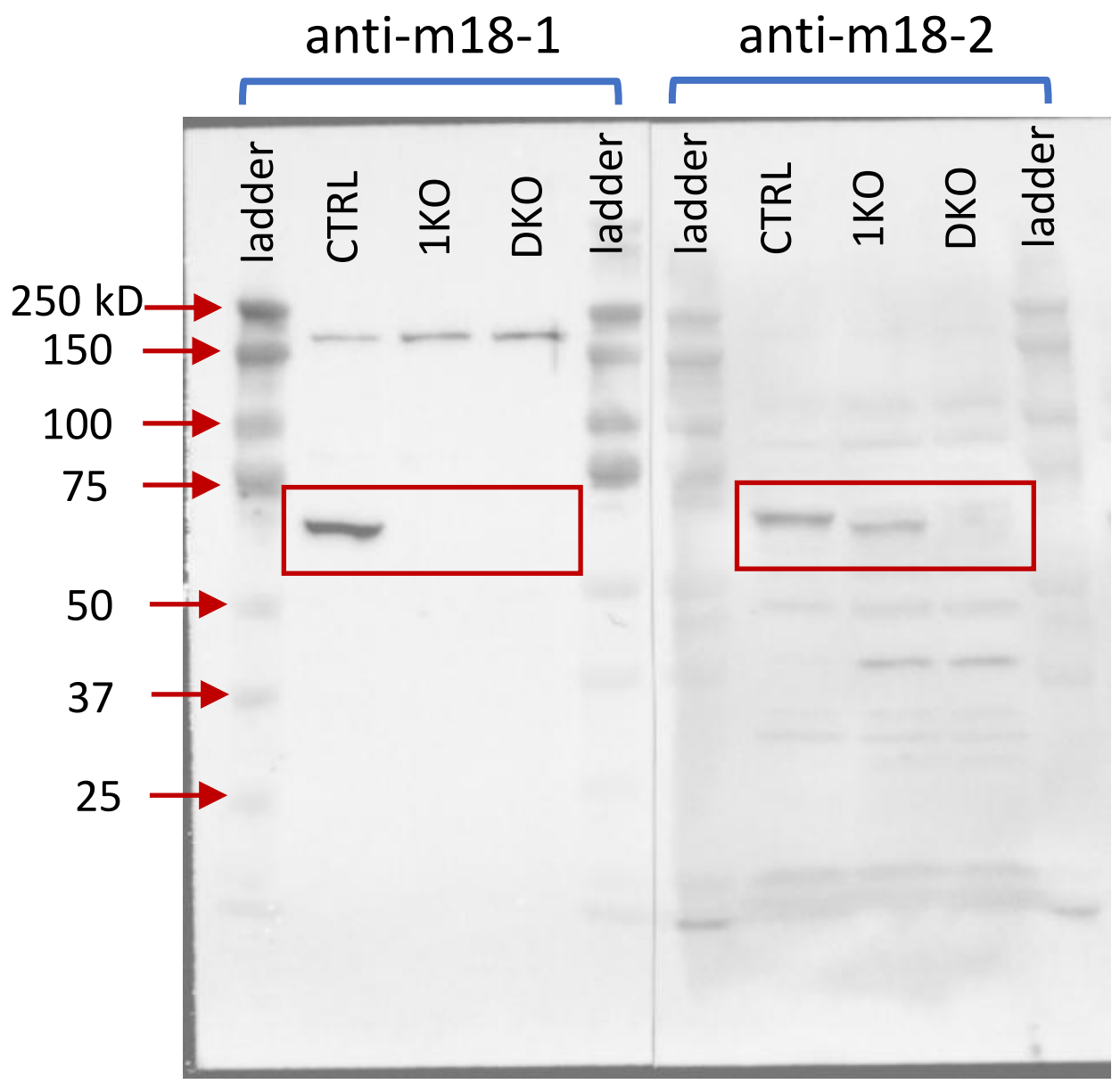

Colorimetric

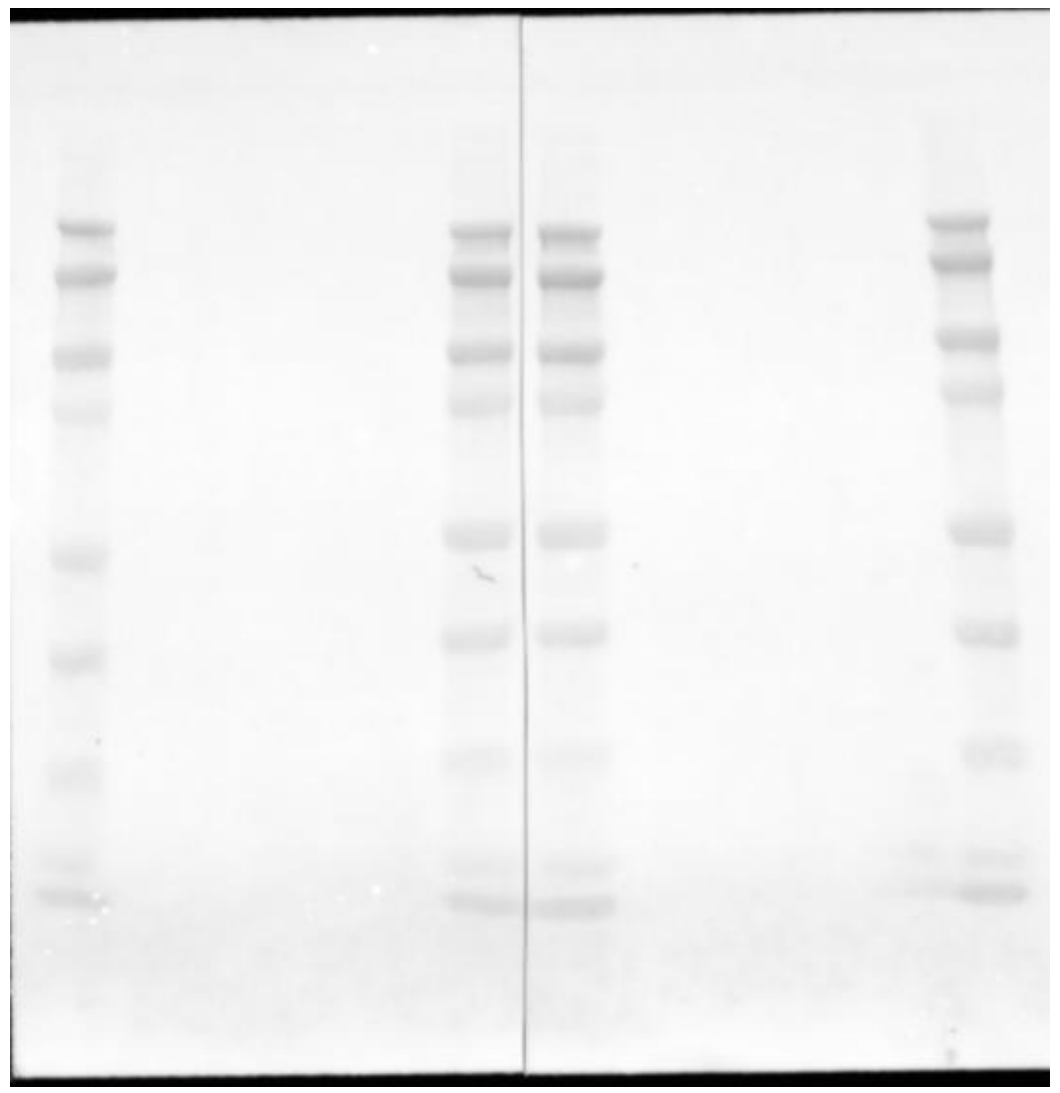

Fig S3A_western blot

Stain free gel (10%)

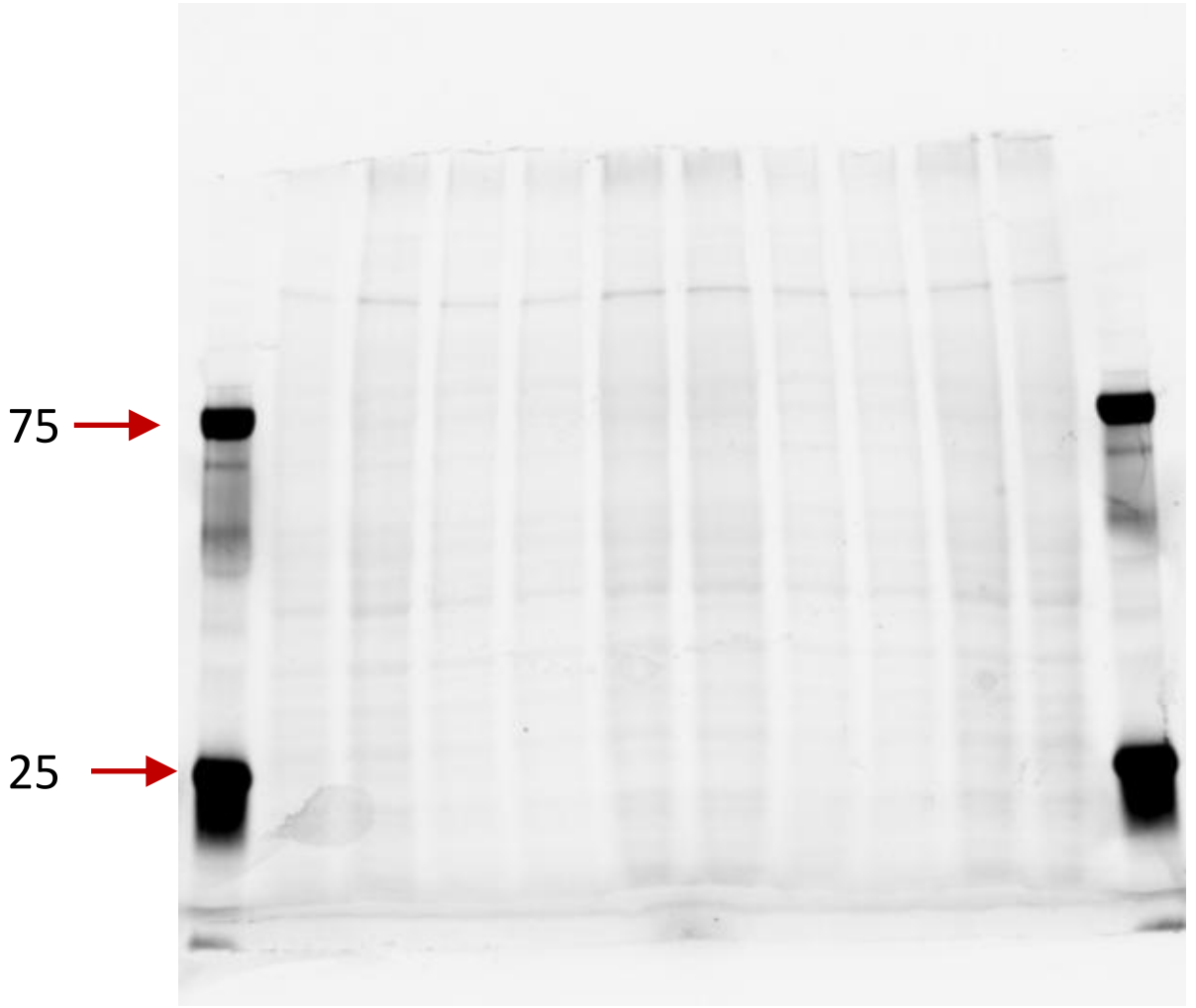

Chemiluminescence

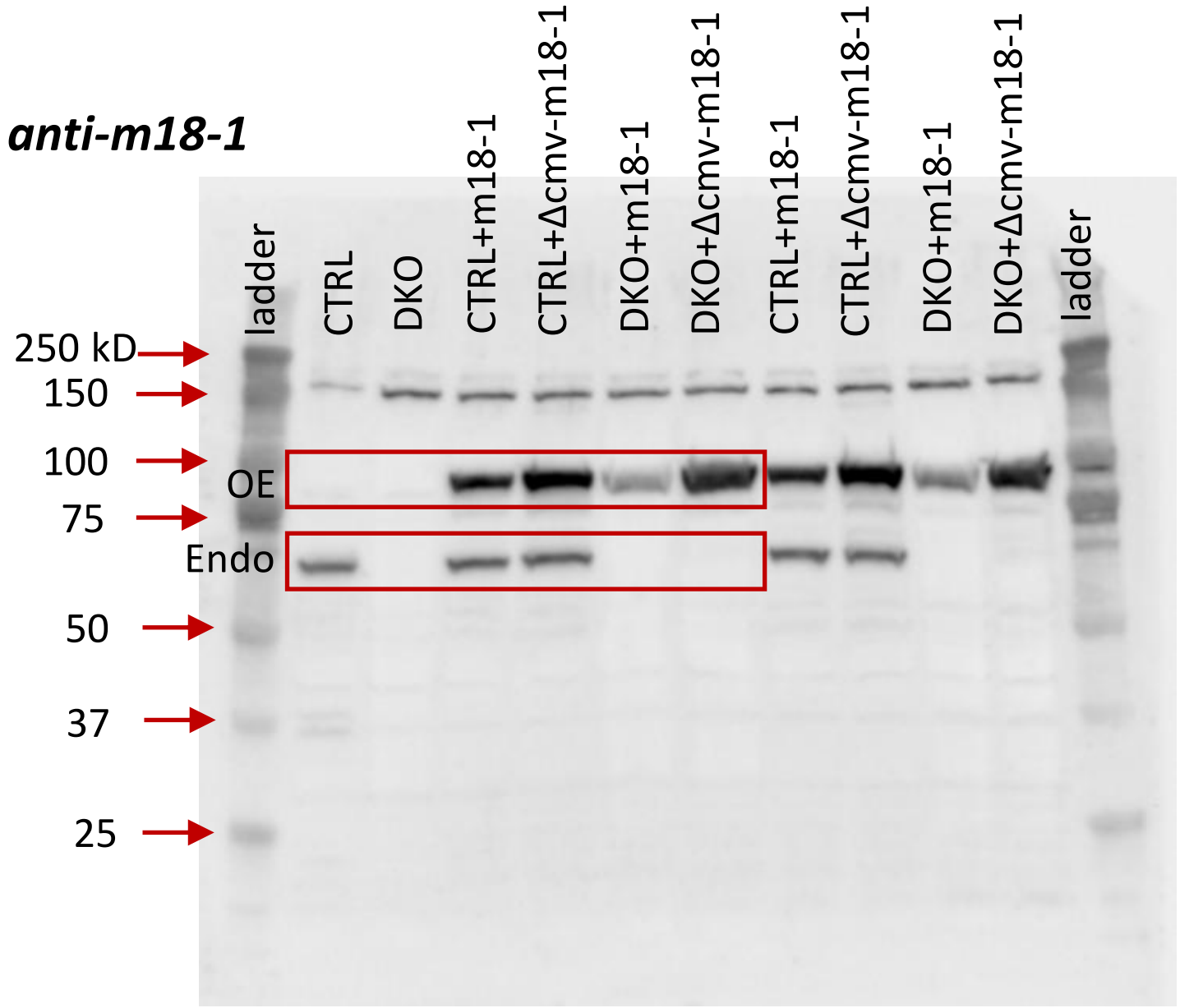

Colorimetric

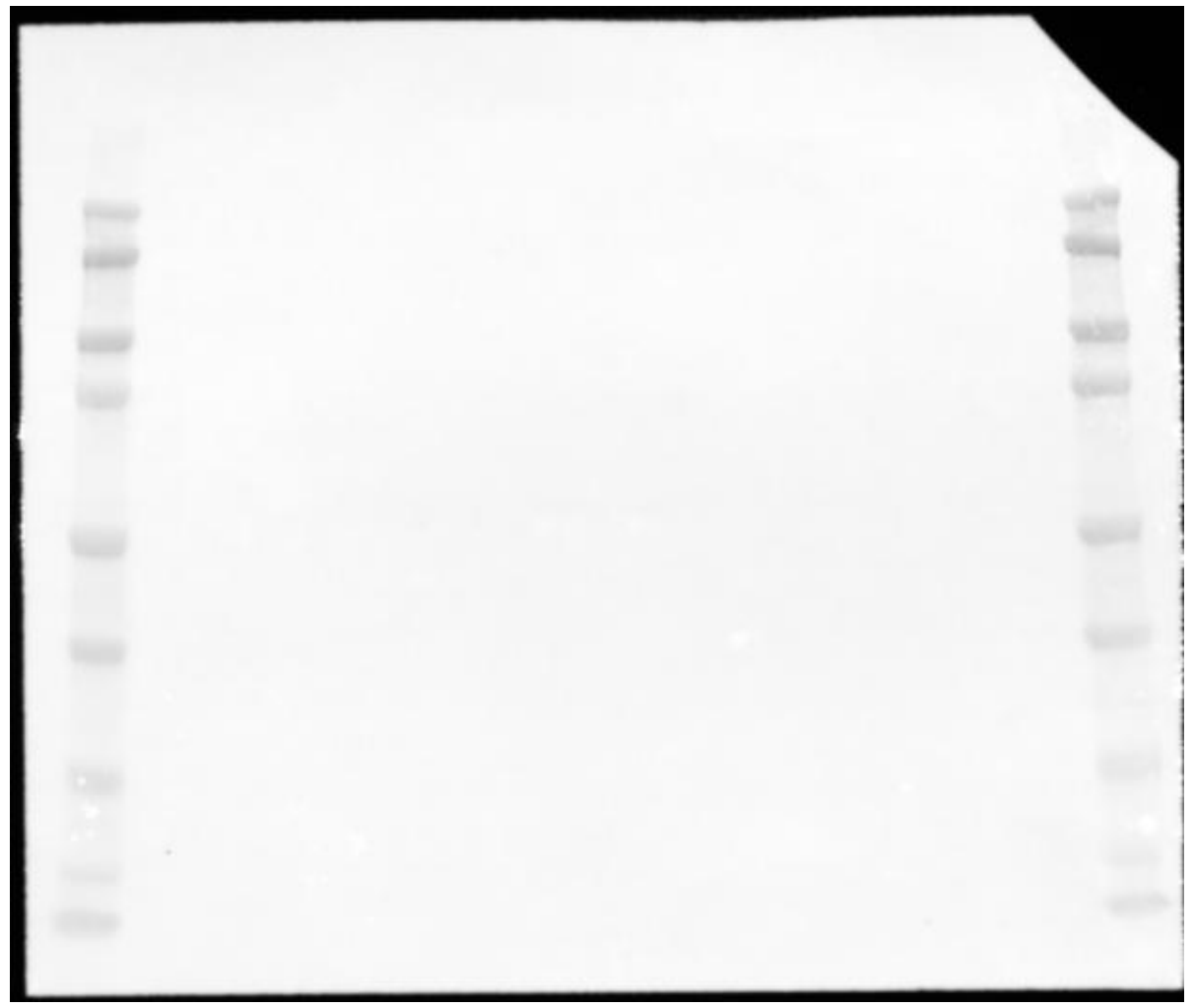

Fig S3C_western blot

Stain free gel (10%)

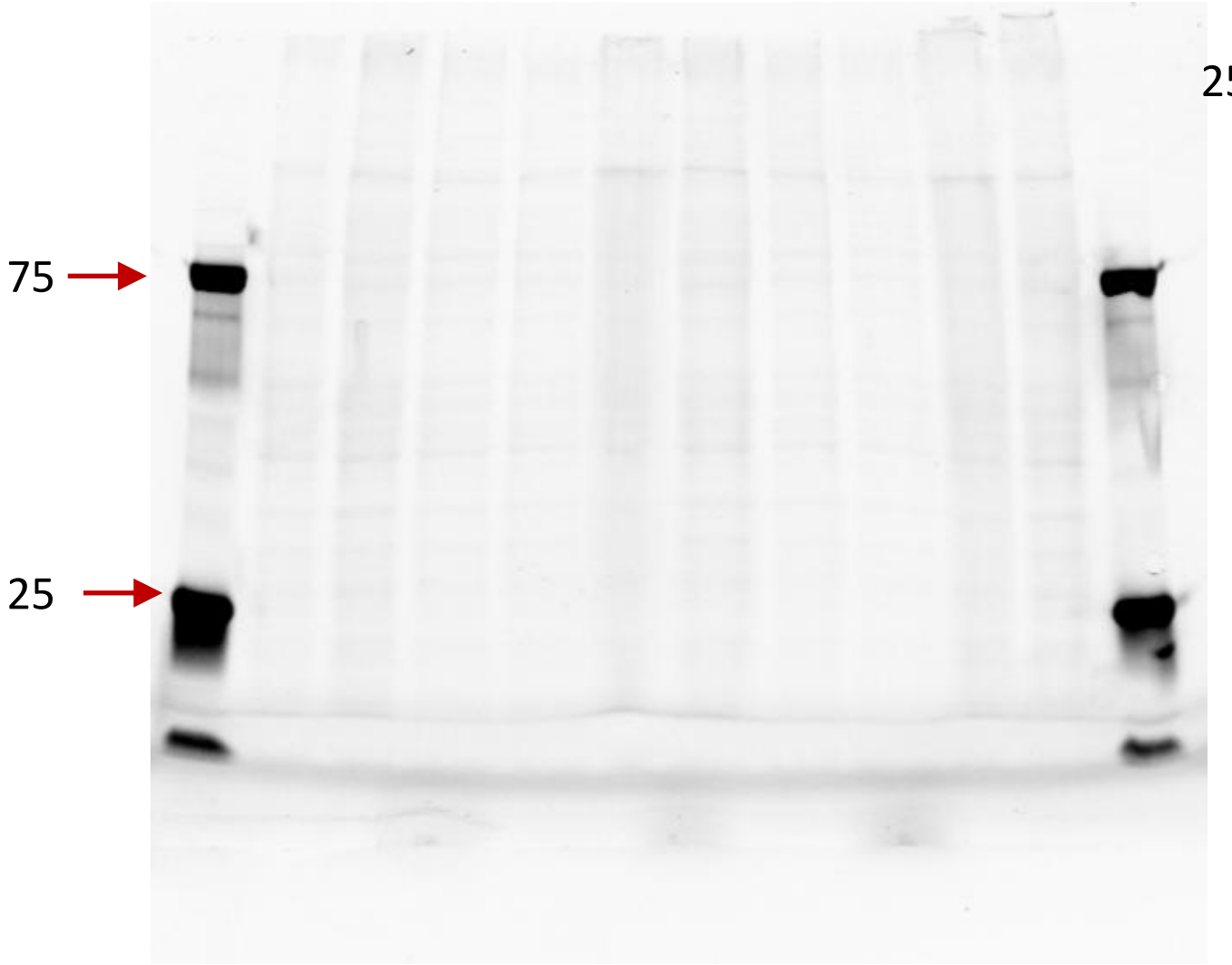

Chemiluminescence

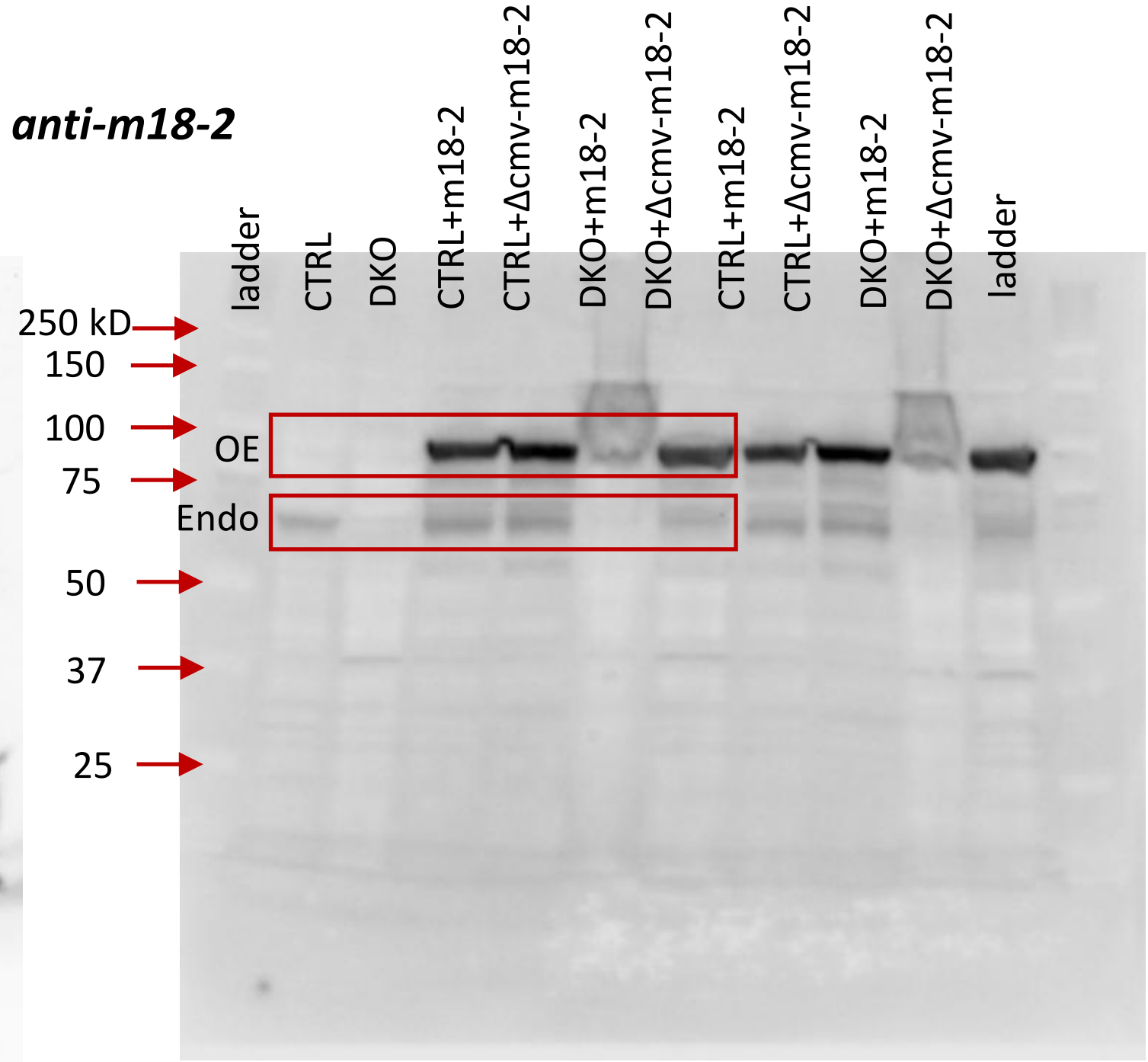

Colorimetric

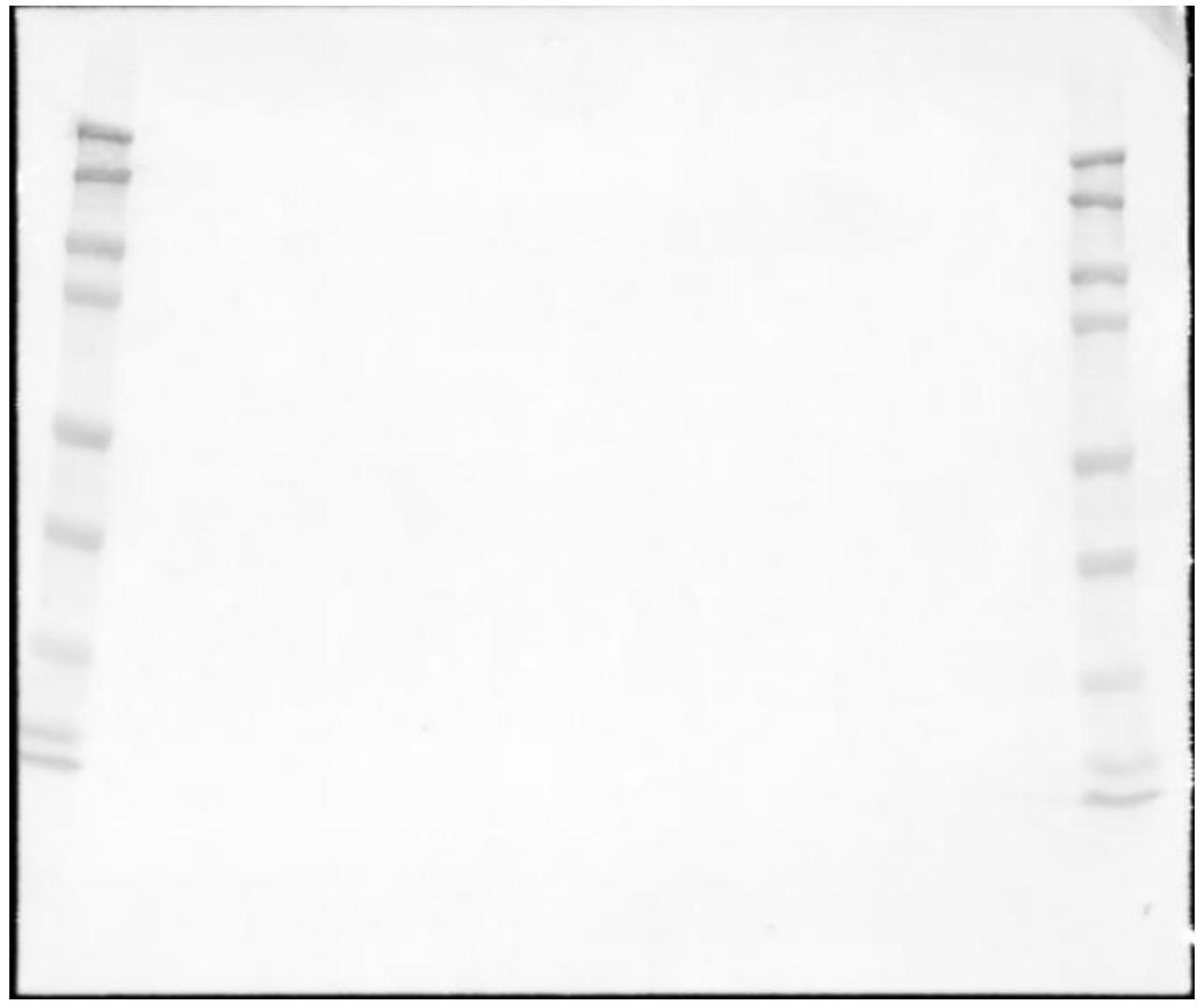

Supplement: Supplementary file 1 [file 18_2026_6381_MOESM7_ESM.pdf]
